# Quantifying Neural Oscillatory Synchronization: A Comparison between Spectral Coherence and Phase-Locking Value Approaches

**DOI:** 10.1371/journal.pone.0146443

**Published:** 2016-01-08

**Authors:** Eric Lowet, Mark J. Roberts, Pietro Bonizzi, Joël Karel, Peter De Weerd

**Affiliations:** 1 Department of Psychology and Neuroscience, Maastricht University, Maastricht, The Netherlands; 2 Department of Knowledge Engineering, Maastricht University, Maastricht, The Netherlands; 3 Donders Institute for Brain, Cognition and Behaviour, Radboud University Nijmegen, Nijmegen, The Netherlands; Federal University of Rio Grande do Norte, BRAZIL

## Abstract

Synchronization or phase-locking between oscillating neuronal groups is considered to be important for coordination of information among cortical networks. Spectral coherence is a commonly used approach to quantify phase locking between neural signals. We systematically explored the validity of spectral coherence measures for quantifying synchronization among neural oscillators. To that aim, we simulated coupled oscillatory signals that exhibited synchronization dynamics using an abstract phase-oscillator model as well as interacting gamma-generating spiking neural networks. We found that, within a large parameter range, the spectral coherence measure deviated substantially from the expected phase-locking. Moreover, spectral coherence did not converge to the expected value with increasing signal-to-noise ratio. We found that spectral coherence particularly failed when oscillators were in the partially (intermittent) synchronized state, which we expect to be the most likely state for neural synchronization. The failure was due to the fast frequency and amplitude changes induced by synchronization forces. We then investigated whether spectral coherence reflected the information flow among networks measured by transfer entropy (TE) of spike trains. We found that spectral coherence failed to robustly reflect changes in synchrony-mediated information flow between neural networks in many instances. As an alternative approach we explored a phase-locking value (PLV) method based on the reconstruction of the instantaneous phase. As one approach for reconstructing instantaneous phase, we used the Hilbert Transform (HT) preceded by Singular Spectrum Decomposition (SSD) of the signal. PLV estimates have broad applicability as they do not rely on stationarity, and, unlike spectral coherence, they enable more accurate estimations of oscillatory synchronization across a wide range of different synchronization regimes, and better tracking of synchronization-mediated information flow among networks.

## Introduction

Neuronal oscillatory synchronization refers to the presence of a more or less fixed relationship among oscillatory modulations in neural activity in different neurons or neuronal populations. Synchronization is widespread in the nervous system [1–3] and it has been suggested to play a role in the coordination of spiking activity across neuronal populations [4–6]. In particular, the phase synchronization [7] of local or distant oscillatory neuronal populations has received substantial scientific interest [8–17] and is central to influential theories like ‘Communication through coherence’ (CTC, [8,18]) and the ‘Binding by synchrony theory’ [19,20]. It is hypothesized that oscillatory phase-locking (phase-consistency) between two brain regions optimizes information transmission [21].

A common property of neural network oscillations is that the spike probability of neurons is influenced by oscillation phase [3,22]. Oscillation cycles define periods of higher excitability where neurons spike and are more sensitive to incoming spikes and periods of lower excitability where spike probability is low and incoming spikes have lower impact [18,23,24]. If two networks can align the oscillatory cycles over time such that the excitability periods are in ‘good’ alignment [18,25], then ‘communication’ (information flow) will be optimized. Given the potential role of oscillatory phase locking for understanding neural information processing, it is therefore critical to have a valid and robust approach for experimentally measuring phase-locking between neural oscillations. Phase-locking is defined here as the amount of consistency between the instantaneous phases of oscillations, or put differently, how ‘peaky’ (non-uniform) the instantaneous phase-relation distribution is.

In the field of neuroscience, phase locking strength is often estimated by computing spectral coherence. For example, in experimental neuroscience, spectral coherence has been frequently used to detect and quantify coupling between oscillatory signals of two or more brain areas [8,12–15]. Spectral coherence estimates the linear phase-consistency between two frequency-decomposed signals over time windows (or trials) [26]. Specifically, spectral coherence reflects the consistency (mean resultant vector length) of cross-spectral densities between two signals, normalized by their auto-spectra densities.

Spectral coherence has a long history [26] and has proven to be a useful method for many scientific questions. Great advantages of spectral coherence are that it is well understood and studied, computationally fast, relatively robust against noise and allows an easy overview over relevant coherent frequencies in the data. However, spectral coherence relies on several assumptions regarding the analyzed signal, with the requirements of linearity and stationarity in the signal being of prime importance. The assumption of (weak-sense) stationarity means that the autocorrelation structure of a signal is not dependent on the reference time point [27]. The Fourier transform assumes that the signal is linear; i.e. that it can use a linear superposition of trigonometric functions to represent it [28]. If these assumptions are violated, spectral coherence might give unreliable estimates. Unfortunately, these critical assumptions are rarely tested in neuroscience studies and it is uncertain how often the violation of stationarity and linearity assumptions of the signal affects the reported coherence results. In addition, there is increasing evidence that neural oscillatory signals show properties of non-stationarity that make them almost by definition unsuited for analysis by spectral coherence methods [10,29–34].

There are various alternative approaches to spectral coherence, which do not rely on the strict assumptions underlying spectral coherence, and which estimate phase-locking (mean vector length) on the reconstructed instantaneous phases [34,35], referred to as the phase-locking value (PLV) approach [34]. These methods might be more suitable for neural oscillatory synchronization as they can better deal with non-stationary (frequency-varying) dynamics. The first aim of the present study was therefore to study the validity and accuracy of spectral coherence to estimate phase-locking in a range of oscillatory synchronization regimes, and to test whether it can robustly track phase-locking dependent changes in information flow between networks. The second aim was to compare spectral coherence with the PLV approach. For the reconstruction of the instantaneous phase we used Hilbert-Transform (HT) preceded by a singular spectrum decomposition (SSD) of the signal into interpretable oscillatory components [36].

To test the behavior of spectral coherence and PLV, we generated testing data, by simulating oscillatory signals with synchronization dynamics [7] using interacting phase-oscillators [37] as well as interacting excitatory-inhibitory spiking neural networks [38,39]. The resulting signals were representative for ordinarily recorded neural signals in neurophysiological studies [40]. Using signals generated by the mathematically simpler phase-oscillator model allowed us to understand the properties of spectral coherence in terms of mathematical properties. In the model, we manipulated the phase-locking strength between the oscillations by changing their detuning parameter (initial frequency difference). The data generated by the more complex neural network model reproduced the synchronization behaviors observed in the phase-oscillator model. On the simulated data, we tested to what extent coherence and PLV were able to capture the (instantaneous) phase relationships among simulated oscillations as well as information transfer among spiking neural networks.

We found that spectral coherence, used here with settings common for neuroscientific research [41,42]deviated, from the expected phase-locking and did not converge to the expected value with increasing signal-to-noise ratio (SNR). The deviation was particularly strong when oscillators were not completely synchronized to 1, either because of detuning (intrinsic frequency difference) or intrinsic noise fluctuations. Moreover, spectral coherence was sensitive to phase-relation dependent amplitude fluctuations showing that it is not a pure phase-locking measure (even when amplitude correlation is 0). These deficiencies of spectral coherence reflect a mismatch between its underlying assumptions and the simulated data, which we suggest is likely to often also affect the usefulness of coherence when applied to experimental data. The PLV approach did not show these drawbacks and it converged to the expected value with increasing SNR. In addition, we used transfer entropy [43] to measure how well spectral coherence and PLV reflected changes in information flow between spiking neural networks. We found that spectral coherence did not robustly reflect changes in information flow between oscillating networks, whereas the PLV did. In summary, these results suggest that spectral coherence should be applied with prudence to neural oscillatory synchronization data, whereas PLV methods relying on the estimation of instantaneous phase appear to provide a more promising approach.

## Methods

### Theory of phase synchronization

Phase synchronization is the process in which oscillators adjust their rhythms [7], a phenomenon that has been first described by Huygens in the 17^th^ century for pendulum clocks [44]. Phase synchronization means that the oscillators have a preferred phase-relation to each other and that the oscillators adjust their phases as a function of their phase difference. The phase adjustment is defined by the ‘phase response curve’ (PRC), that has been described in various neuroscience domains [45]. The PRC captures the mutual forces that coupled oscillators exert on each other depending on their phases. The PRC thus defines how much a given force exerted by one oscillator at a given phase will delay or advance another oscillator’s phase, as a function of the latter oscillator’s phase. Hence, the PRC also defines which phase-relations among oscillators occur preferentially, thus representing fixed attractor points in the phase-relations among oscillators. The Theory of Weakly Coupled Oscillators (TWCO) [37,46–48] describes mathematically the phase dynamics among weakly interacting oscillators. ‘Weak’ means that interactions lead to phase adjustments without strong perturbations of the oscillatory generative mechanism. ‘Strong’ coupling can lead to chaotic regimes [49,50] or to ‘oscillation death/quenching’ [7,51]. The TWCO has been applied in many neuroscience fields, including in gamma-generating neural network models [52]. To simulate oscillatory synchronization data, we used a basic model of phase-oscillators [37] that is simple, yet exhibits plausible phase synchronization dynamics relevant for neuroscience.

**Fig 1:**
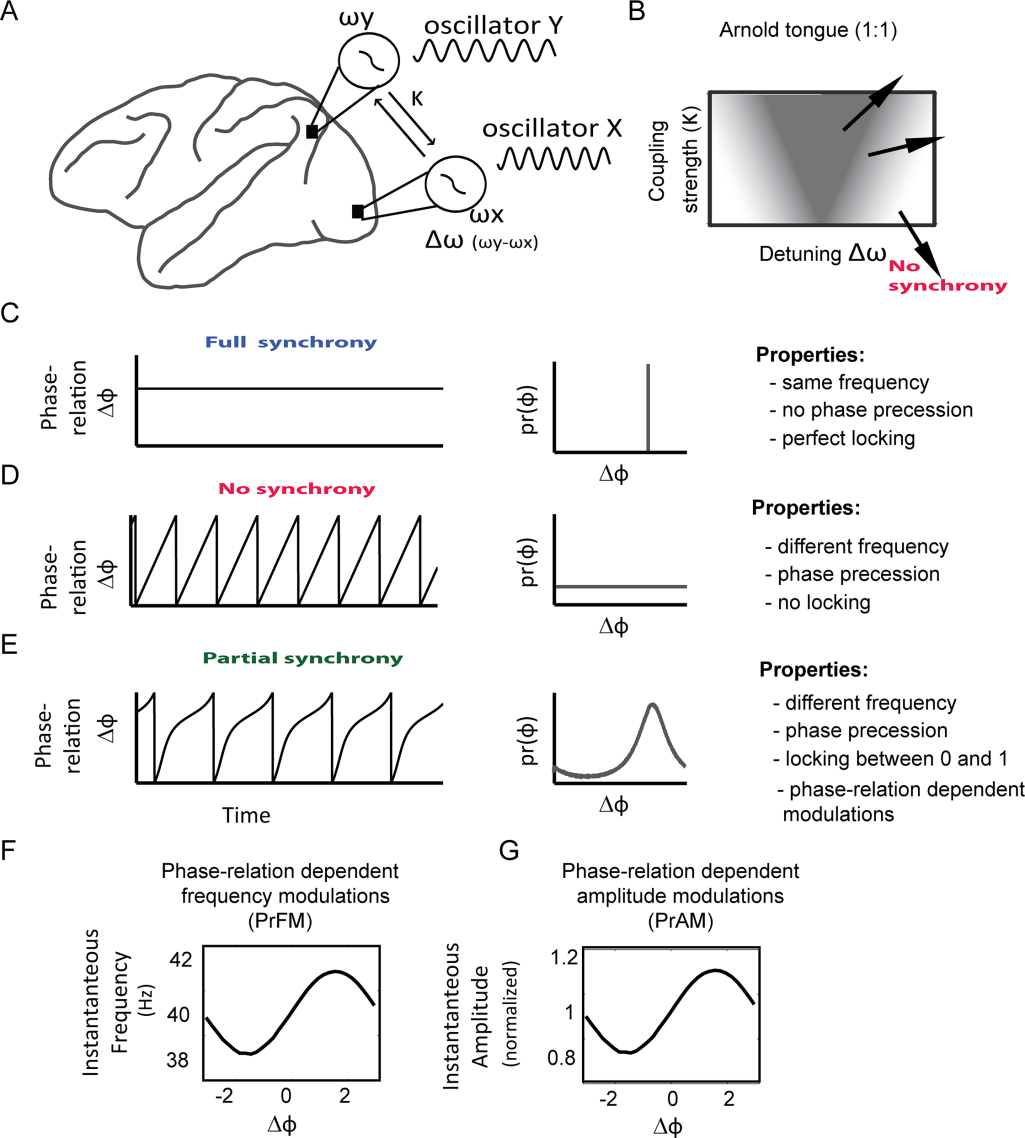
Basic properties of oscillatory phase synchronization. (A) The underlying model of this study was phase synchronization of two coupled phase-oscillators which could correspond to oscillatory signals measured from separate cortical areas. Phase-locking is the amount of consistency of instantaneous phases between the two oscillators. Phase-locking is resulting from synchronization process governed by two principal factors as described by Theory of Weakly Coupled Oscillators (TWCO): The intrinsic (natural) frequency ω and the coupling strength κ. The intrinsic frequency difference (detuning Δω) between oscillators determines the phase precession. The coupling strength κ determines the interaction strength, which is a function of the phase-relation (defined by the phase response curve, PRC). (B) The detuning Δω and the coupling strength κ defined a 2-dimensional space, in which phase-locking (gray shading) occurs within certain ranges. In a noiseless system, full synchrony (phase locking of 1) occurs in a limited area of detuning and coupling strength that appears as inverted triangle (Arnold tongue). The stronger the coupling strength, the more detuning is possible while still reaching full synchrony. Full synchrony (C) occurs if the oscillators converge on a common frequency (no phase precession). The phase-relation distribution exhibits a strong peak at the attractor phase relation. (D) Complete asynchrony is only possible when the oscillators are uncoupled. The phase-precession is smooth and the phase-difference distribution uniform. (E) The state of partial synchrony is characterized by phase-locking between 0 and 1. In most regions in the Δω vs. κ space, phase-locking might be close to 0. Yet, close to the Arnold tongue the phase-locking might still be relevant. The oscillators do not converge to a common frequency, but exhibit phase precession. The phase precession does not have a smooth trajectory, but is modulated depending on the phase-relation. This leads to non-uniform phase-difference distribution with a peak at the phase-relation in which the oscillators have the smallest frequency difference. In noisy phase-oscillatory systems, the partial synchronized regime can be the most dominant regime. (F) Because the phase-precession (= instantaneous frequency difference) is not smooth and changes as a function of phase-relation, it implies phase-relation dependent frequency modulations (PrFM). (G) We also included phase-relation dependent amplitude modulations (PrAM) due to observations in many of our neural network simulations. We assume here that PrAM, in the ranges included here, did not substantially change the phase trajectories and hence TWCO is still an adequate theoretical framework.

The synchronization properties of two coupled phase-oscillators X and Y (Fig 1A) are governed by two factors: the level of detuning Δω (intrinsic or natural frequency difference, Δω = ω_X_—ω_Y_) and the coupling strength κ. The detuning Δω determines the phase precession frequency (de-synchronization force) and the coupling κ determines the strength of phase adjustments (synchronization force). Both parameters define a two-dimensional space in which the phase-locking between oscillators can be determined. In this space one can observe inverted triangles that define the phase-locking region in the detuning versus coupling space. Such triangular phase-locking region looks like a tongue, and is referred to as the ‘Arnold’ tongue [7] (Fig 1B). The triangular shape derives from the fact that oscillators with stronger coupling strength *κ* can converge to a phase-locking state for larger detuning values Δω.

Phase-locking can be formally defined as the constancy of the instantaneous phase-relation between oscillators (Fig 1C), which hence show no phase precession [7]. This means that instantaneous phase of one oscillator always perfectly predicts instantaneous phase of the other, in which case phase-locking has a value of 1. A lower phase locking value represents the lower probability of making this prediction correctly. The mathematical definition of phase-locking is given in Eq 1.

$$|n\varphi_{Y}\left(t\right)-m\varphi_{X}\left(t\right)|=c\forall t,\;c\in R,\;m,n\in Z\;.$$ (1)

The variables *n* and *m* are integers and represent the frequency ratios (1:2,2:3,…, [7,52]) in which the oscillators can satisfy the condition of phase-locking despite different frequencies ω_X_ and ω_Y_. Therefore one can observe several (higher-order) Arnold tongues. Here, we focused on phase-locking with *n = m = 1* (Arnold tongue 1:1). This is because we were interested in the quantification of phase synchronization between oscillators with nearby frequencies (oscillators within a ‘frequency band’, e.g. the gamma band).

If ω_X_ ≠ ω_Y_ (different frequency) and coupling strength *κ* = 0 (no synchronization), then the phase-relation distribution is uniform (full phase precession, see Fig 1D). Yet, phase-locking is often neither completely synchronous nor completely asynchronous. The incomplete phase-locking is called the partially synchronized state [7] in which oscillators exhibit a preference for particular phase-relations intermixed with periods of phase precession (Fig 1E). This implies that phase-locking can be of different magnitudes. This is in line with the idea that biological systems are inherently noisy and time-varying (i.e. variable frequencies over time) and thus unlikely to engage in complete synchrony.

The fact that the partially synchronized state is characterized by incomplete phase-locking and therefore phase precession entails that oscillators in this state will traverse all possible phase-relations over time. As we have described above, the PRC defines the phase adjustments in terms of positive or negative delays as a function of phase-relation and time. The rate of phase change over time, which is the time derivative of phase, defines instantaneous frequency (IF) [7]. Close to the preferred phase-relation, the IF difference between phase-oscillators is minimized (phase precession slows down), whereas in non-preferred phase-relations the IF difference is maximized (phase precession is faster and IF approaches the intrinsic frequency). This will lead to systematic phase-relation-dependent IF fluctuations (PrFM) (Fig 1F). The main frequency of PrFM equals the phase precession frequency [7]. In addition to PrFM, there can also be a form of phase-relation dependent amplitude modulation (PrAM). In our raw simulations, according to the TWCO, the phase-oscillators had unit amplitude, which did not change during the synchronization dynamics. However, we included PrAM post-hoc to the simulated oscillatory signals (Fig 1G), based on our observation of PrAM in simulations of interactions between gamma-oscillation generating excitatory-inhibitory neural networks during partial synchronization (see below).

The phase-relation dependent frequency modulations (PrFM) in our simulation of coupled phase-oscillators can be described with the following equation:

PrFM(φ)=κPRC(φ) (2)

where ϕ is the phase-relation between the two oscillators, PRC is the phase response curve defined here as a sinusoid, and *κ* the coupling strength. Hence, the modulation function of PrFM is directly related to the PRC, whereas the strength of the fluctuation depends on the coupling strength *κ*.

The phase-relation dependent amplitude modulations (PrAM) were defined as:

PrAM(φ)=αcos(φ) (3)

where ϕ is the phase-relation between two oscillators, cos is the cosine modulation function (maximal amplitude at phase 0), and α is the amplitude modulation strength. Note that α was defined as percentage modulation of oscillation amplitude. For example, a α of 20% modulation means that the oscillation amplitude varies by 20% as a function of phase (e.g. for an amplitude of 1 the means a variation between 0.8 and 1.2).

### Generative models of oscillatory synchronization

#### Oscillations generated by the phase-oscillator model

For generating testing data with plausible and well-understood synchronization properties, we used the phase-oscillator model as the underlying generative model. The model is very similar to the well-known Kuramoto model [37]. Here, we simulated two zero-mean oscillatory signals X(t) and Y(t) governed by the phase-oscillator equation. We restricted ourselves for simplicity to the case of unidirectional coupling (X→Y). However, all results can be generalized to the case of mutually coupled phase-oscillators.

The phase evolution ϕ_x_(t) of oscillator X, unperturbed by oscillator Y, is defined only by its intrinsic frequency ω_x_ and an intrinsic phase noise process Np_x_ (if included):

$$\frac{d\varphi_{X}\left(t\right)}{dt}=\omega_{X}\left(t\right)+{\text{Np}}_{X}(t)$$ (4)

In case of oscillator Y, the exact phase evolution ϕ_x_(t) depends on the interaction term (phase response curve, PRC) with oscillator X and the coupling strength κ:

$$\frac{d\varphi_{Y}\left(t\right)}{dt}=\omega_{Y}\left(t\right)+\kappa \text{sin}(\theta \left(t\right))+{\text{Np}}_{Y}(t)$$ (5)

with θ(t) = ϕ_y_(t)–ϕ_x_(t). The interaction term describes the phase adjustments (phase response curve, PRC) induced by the other oscillator X depending on the coupling constant κ. As the interaction term we used a sinusoidal function with an attractor fixed point (in-phase). It has been shown that the evolution of the phase relation between the two oscillators without intrinsic noise (Np = 0) can be described with a single equation, referred to as the Adler equation [7]:

$$\frac{d\theta (t)}{dt}=\Delta \omega (t)+\kappa \;*\;\text{sin}\left(\theta (t)\right)$$ (6)

The equation shows that the time evolution of the phase relation θ(t) is a function of the frequency difference Δω(t) at that time, as well as the coupling strength *κ* of the sinusoidal interaction function. In case of Np = 0 (no intrinsic noise), the phase-locking properties of the two coupled phase-oscillators can be analytically derived using the Adler equation (Methods A in S1 File). As described above, the phase-locking properties are a function of the independent variables detuning Δω and coupling term κ. This means that depending on the chosen parameter ranges for Δω and coupling term κ, phase-locking can vary between 0 and 1 in a manner that is fully deterministic (as long as the phase oscillator model is noise-free).

Hence, for a given parameter set used for simulating data, we generated the instantaneous phase traces by numerically solving the differential equations (Euler method, step size 1ms). We generated 500 trials of 3sec simulation with randomized initial phase conditions and intrinsic frequencies in the gamma frequency range (30-50Hz). The frequency range is of no importance for the phase-oscillator model and the results can be generalized to any frequency range. For each trial the first 2sec were discarded to exclude any transient dynamics and to have a state as stable as possible. In one exceptional case we used 200ms time windows instead of the 1s time window after the first discarded 2sec. The (instantaneous) phase traces were wrapped around ±pi. We generated multiple shorter trials instead of a long single trial to permit easy application of spectral coherence.

As a next step, we converted the phase-traces into zero-mean real-valued signals to be used for testing phase-locking estimation methods. In experimental measurement conditions, the signals always suffer from added extrinsic (measurement noise) which is signal unrelated to the process of interest. We therefore added different levels of zero-mean extrinsic white noise. The amount of signal-to-noise ratio (SNR) was defined as defined as:

$$SNR\left(\omega \right)=\frac{S_{\text{SN}}{\left(\omega \right)}^{2}}{S_{\text{N}}{\left(\omega \right)}^{2}}-1$$ (7)

where S_SN_(ω)^2^ stands for spectral power of the noisy oscillatory signal at frequency *ω* and S_N(_ω)^2^ stands for spectral power of just the noise term. This is also the definition of the relative power ratio. In some conditions, we added phase-relation dependent amplitude fluctuations (PrAM) in oscillator Y using Eq 3. The amount is defined as amplitude modulation in % of the mean oscillator amplitude.

#### Oscillations generated by spiking excitatory-inhibitory neural network model

To demonstrate that the results from the phase-oscillator equations are generalizable to more biophysically plausible neuronal network oscillations, we simulated two coupled excitatory-inhibitory spiking neural networks generating pyramidal-interneuron gamma (PING,[39]) oscillations.

The neural voltage dynamics *v* were of the Izhikevich-type [38] and defined as follows:

$$\frac{dv}{dt}=0.04v^{2}+5v+140-u+I$$ (8)

$$\frac{du}{dt}=a\left(bv-u\right)$$ (9)

$$if\;v\geq 30mV,\;\text{then}\;\{\begin{array}{c} v\leftarrow c\\ u\leftarrow u+d \end{array}$$ (10)

The coupled differential equations were numerically solved using the Euler method (1ms step size). We simulated 300 trials of 1.3sec length, from which the first 300ms were discarded to reduce transient dynamics. The networks were both composed of two types of neurons: 400 regular spiking neurons RS (a = 0.02,b = 0.2, c = -65mV, d = 8) and 100 fast-spiking interneuron FS (a = 0.1,b = 0.2, c = -65mV, d = 2). RS were excitatory neurons and FS inhibitory neurons (ratio 4:1). The neural networks were all-to-all synaptically connected. Synapses were modeled as exponential decaying functions, reset to 1 after the presynaptic neurons fired. Synaptic connection values set the maximum synaptic connection strength (max syn). The synaptic strengths were chosen from a random uniform distribution defined between the 0 and the maximal connection strength.

Within a network, RS neurons projected excitatory synaptic AMPA (decay constant = 2ms) connections onto FS neuron (max syn = 0.45mV) and among themselves (max syn = 0.05mV). FS neurons projected synaptic GABA-A (decay constant = 8ms) connections onto RS neurons (max syn = -0.35mV) and among themselves (max syn = -0.2mV). For cross-connections between network 1 and 2, we included RS→FS connections (E→I_,_ max syn(default) = 0.015mV) as well as RS→RS connections (E→E_,_ max syn(default) = 0.007mV). We included a conduction delay of 1ms for all connection types.

The input drive to RS neurons was composed of a fixed input current to each neuron (10mv), unique Gaussian input noise for a given neuron (SD±3.5mV) and a 1/f^1.5^ input noise shared among neurons (±3.5mV std) of the same network. So each network received uncorrelated 1/f^1.5^ input noise to RS neurons with the effect to induce instantaneous frequency variation of a network over time (similar to intrinsic phase noise in the phase-oscillator model). For FS neurons, each received a fixed input current (3.5mV) and Gaussian input noise (SD±3.5mV). FS neurons received further excitatory drive from the RS neurons.

For reconstructing a population field signal for both networks (resembling LFP signals), we summed the spike trains of all RS neurons for a given network, followed by demeaning and smoothing with a pseudo-Gaussian kernel (SD = 3ms). For computing the expected PLV, we Hilbert transformed the signals and computed their instantaneous phase-relations from which the PLVs were derived. For the generation of the final testing data, we added different levels of extrinsic noise (here: 1/f^1.5^ noise).

### Phase-locking estimation methods

Was assumed that signals X(t) and Y(t), which might represent two cortical regions, contained underlying oscillatory processes with instantaneous phase evolution ϕ_X_(t) and ϕ_Y_(t). We were interested to understand how interdependent or phase-locked the two phase parameters were. Or in other terms, how consistent the phase-relations θ(t) = ϕ_X_(t)—ϕ_Y_(t) were over time. If one measures the occurring phase-relation θ(t) for a period T, one obtains a distribution of phase-relations. For quantifying the consistency of phase-relation θ(t), we computed the mean resultant vector length (MRVL). Each phase can be represented as a vector in the complex plane. If θ(t) is consistent over time, the vectors have the same angle, therefore the vectors add up and the MRVL will be non-zero. If vectors are unity (= 1) then the MRVL will be 1. If θ(t) is not consistent, the vectors will be equally distributed over–π to π and will cancel each other out. The MRVL will be 0. The MRVL is appropriate if the ϕ_X_(t) and ϕ_Y_(t) are linearly interdependent (assumed here).

In this study the MRVL for phase-locking estimation was applied on phase-relation distributions obtained by two very different approaches. The first one was based on the normalized Fourier cross-spectral coefficients which is referred to as the ‘spectral coherence approach’ [26]. The second was based on the estimation of the instantaneous phases using Hilbert Transform or time-frequency representations and is termed the ‘phase-locking value (PLV)’ approach [34] (see Figure A in S1 File for schematic illustration of the approaches). Both approaches are used currently in neuroscience. In this study we test explicitly whether the two approaches are appropriate phase-locking estimation methods for oscillatory synchronization. In the following segment we introduce the spectral coherence and the PLV approaches in more detail. Note that the reason we limited ourselves to a description of two specific methods is because we did not aim to make a detailed investigation of the performance of different variations of spectral coherence and different variations of the PLV approach (e.g., testing different signal decomposition methods or different approaches for extracting instantaneous phase). We suggest that the findings we will report here for the specific spectral coherence and the specific PLV approach used here will be representative, respectively, for the classes of methods computing phase relations based on spectral methods, and those computing phase relations based on instantaneous phase estimations.

#### Spectral coherence

Spectral phase-locking measures are used in many experimental studies and also offered by widely-used analysis toolbox in neuroscience as the principal method to quantify phase-locking (Fieldtrip [41], Chronux [42]). Commonly used spectral-based measures are the coherence index [26] or its modification [53] to increase robustness against amplitude correlation. Here, the time-domain signals of each trial are transformed in the frequency-domain and phase-coupling is assessed frequency-by-frequency. The advantage is that one can observe, in a computationally efficient manner, frequency-resolved peaks in phase-locking, which yield a quick overview of phase locking over relevant frequencies. However, the spectral coherence measure assumes (weak-sense) stationary processes.

To estimate the spectral phase, we computed the discrete Fourier transform of the time series of the oscillatory signal X(t,n) (t = 0,…T-1) of a given trial n with length T.

$$S_{x}\left(\omega ,n\right)=\sum_{t=0}^{T-1}X\left(t,n\right)e^{-i2\pi \omega t/T}$$ (11)

where S_x_(ω) is the complex-valued Fourier coefficient at integer *ω* related by *ω* / *T* to normalized frequency. For simplicity we will call *ω* just frequency assuming appropriate rescaling. The power spectrum is defined as:

$$S_{xx}\left(\omega ,n\right)=E[S_{x}\left(\omega ,n\right)S_{x}{\left(\omega ,n\right)}^{*}]$$ (12)

where S_xx_(ω) is the estimated spectral power of oscillation X at frequency ω, E[] is estimation of a function and * the complex conjugate. S_YY_ was computed as S_XX_. The spectral coherence between two signals *X(t*,*n)* and *Y(t*,*n)* are based on the cross-spectral density estimate of S_x_(ω,n) and S_y_(ω,n), defined as follows:

$$S_{xy}\left(\omega ,n\right)=E[S_{x}\left(\omega ,n\right)S_{y}{\left(\omega ,n\right)}^{*}]$$ (13)

where S_xy_(ω,n) is the estimated complex-valued cross-spectral density at frequency ω, where E[] is estimation of a function and * is the complex conjugate. The cross-spectral density reflects both the mean phase-difference as well as the power correlation between S_x_(ω,n) and S_y_(ω,n). The spectral (sample) coherence Coh(ω) [53] is the absolute value of the cross-spectral density normalized by the respective power spectra and is defined as follows:

$$\text{Coh}\;\left(\omega \right)=\frac{\left|\sum_{n=1}^{N}Sxy\left(\omega ,n\right)\right|}{\sqrt{\left(\sum_{n=1}^{N}Sxx\left(\omega ,n\right))\;(\sum_{n=1}^{N}Syy\left(\omega ,n\right)\right)}}$$ (14)

where *n* is the trial number (*n = 1*,.*N*) used for estimation. A critical point of spectral coherence is that the S_xy_(ω) value depends on the phase as well as the amplitude correlation. Each trial therefore contributes as a function of amplitude correlation, making spectral coherence sensitive to amplitude correlation values [34,54].To make spectral coherence not sensitive to amplitude correlation the spectral coherence formula, used in the following Result sections, was modified [53] as follows:

$$\text{Coh}\;\left(\omega \right)=\left|\frac{1}{N}\sum_{n=1}^{N}\frac{Sxy\left(\omega ,n\right)}{\sqrt{Sxx\left(\omega ,n\right)Syy\left(\omega ,n\right)}}\right|$$ (15)

Each absolute cross-spectral product for a given trial n and frequency *ω* is normalized with the square-root product of the autospectra S_xx_(ω,n) and S_yy_(ω,n). Therefore different levels of amplitude correlations do not affect the phase-locking measure. It has therefore been assumed that this spectral coherence formula is a pure phase-locking measure [41,53]. It can be seen as applying MRVL on the angle values of the complex Fourier cross-spectral coefficients. This formulation was used to assure straightforward comparison to the other phase-locking estimates used in this study. The results shown in this study for spectral coherence as defined by Eq 14 or Eq 15 can be expected to be very similar, because the amplitude correlations were negligible in our simulations (also if PrAM included). The effect of amplitude correlation on spectral coherence has recently been studied systematically [54].

For a given testing data set we first computed the (Fast) Fourier transform (FFT). For simplifying the derivation of the analytical coherence value we did not use tapering or padding. Then we computed the spectral coherence spectra. We took the maximum (peak height) of the coherence spectrum. The peak of the coherence spectra was the phase-locking estimate, which was compared to the expected phase-locking. We applied trial-number correction formula (see below) for the coherence estimates.

#### Estimation based on the instantaneous phase: phase-locking value (PLV)

Various methods have been proposed that quantify phase-locking based on the instantaneous phase [55]. These methods deal better with non-stationary dynamics, which are likely to be present in neural signals. The main challenge is to decompose the often complex multi-component measured brain signal into well-defined oscillatory components (e.g. through filtering or wavelet decomposition techniques) from which the instantaneous phase can be extracted (i.e., after a Hilbert-Transform or directly from a time-frequency representation (TFR), [35]). Below we propose an alternative method that is based on singular spectrum decomposition (SSD, see https://project.dke.maastrichtuniversity.nl/ssd/) [36] and the Hilbert-Transform. We then applied this method on the simulated two coupled phase-oscillator as well as neural network signals having intrinsic and extrinsic noise.

SSD is a recently proposed method for the decomposition of nonlinear and non-stationary time series [36,56]. In the present work, the method is applied to reduce the influence of noise and to provide a PLV estimate that unlike spectral coherence is able to handle nonstationary signals. Additionally, the SSD method is also able to deal with nonlinear signals unlike wavelet-based approaches. Here, the key ideas underlying SSD are introduced (see [36] for additional details). The method originates from singular spectrum analysis (SSA), a nonparametric spectral estimation method used for analysis and prediction of time series. The advantage of SSA-derived components over Fourier-derived sines and cosines is that SSA-components are model-free (data-driven), and therefore are not necessarily harmonic functions. Being data-driven, SSA components can capture highly non-harmonic oscillatory shapes, making them suitable for the analysis of nonlinear and non-stationary time series. In the SSD method, the choice of the main SSA parameters, the embedding dimension and the selection of the principal components for the representation of a specific component series have been made fully data-driven and automated. This makes SSD an adaptive decomposition method. Similar to empirical mode decomposition (EMD) [57], the decomposition is based on the extraction of the energy associated with various intrinsic time scales. One advantage of SSD over EMD is that it tends to avoid mixing components with different frequency bands and provides accurate separation between intermittent components at the transition points [36].The Hilbert Transform can be used on SSD components when interpreting its outcome with caution. Indeed, SSD-components contain several frequencies, with no clear indication about how many instantaneous frequencies per time instant may be present [57]. However, the narrow-banded frequency content of each SSD-component permits one to consider the results of the Hilbert Transform as sufficiently reliable under most conditions [36].

For both oscillator signals X(t,n) and Y(t,n) we applied SSD for each trial and oscillator separately to extract the oscillatory components (SSD_comp_). For deriving the instantaneous phase of a SSD component, we applied the Hilbert transform (HT).

$$\text{SSD}\alpha_{comp}={\text{SSD}}_{comp}+i\text{HT}\left({\text{SSD}}_{comp}\right)$$ (16)

where HT(SSD_comp_) is the Hilbert-Transform of the selected SSD component. The HT in essence adds the imaginary component to a real-valued signal to reconstruct the analytical signal. SSDα_comp_ is the analytical signal of the SSD_comp_. The instantaneous phase ϕ and frequency *ω* can then easily be derived from the analytical signal:

$$\varphi =\text{arg}\;\left(\text{SSD}\alpha_{comp}\right)\;\text{and}\;\frac{d\varphi }{dt}=\omega$$ (17)

To compute the phase-locking value (PLV), we first computed the instantaneous phase relations θ(t,n) = ϕ_x_ (t,n)–ϕ_Y_(t,n). We then concatenated the trials to compute the overall distribution of phase relations and to eliminate variable n. The PLV was then computed simply as:

$$\text{PLV}=\left|\frac{1}{T}\sum_{t=1}^{T}{\text{e}}^{\text{i}\theta \left(\text{t}\right)}\right|$$ (18)

with T representing here the overall number of sample time points. Eq 18 is the mean resultant vector length (MLVR).

Note that in contrast to spectral coherence, which cannot give single-trial estimates, the estimation of phase-locking for single-trials is possible for the PLV approach [34]. This is interesting if one expects that the synchronization properties (e.g. detuning or coupling strength) change over trials. In all of the work presented in this study, we assume that a given state of (partial) synchronization is constant for the duration of the trial or time window used.

#### Expected phase locking

To determine the expected phase-locking (PL), we computed (without any extrinsic noise) the instantaneous phase-relation between oscillator X and Y. We concatenated all the trials again to have an overall instantaneous phase-relation distribution. From this distribution, we computed the mean resultant vector length. In the case of no intrinsic noise (Np = 0), we also analytically derived the expected phase-locking (Methods A in S1 File). The comparison of the numerically and analytically derived expected PL showed that they were very closely matching (mean square error (MSE) = 1.4e^-5^).

#### Trial-number unbiased phase-locking estimates

To compare the estimates of coherence and PLV in simulated data, we relied on an adapted formula for coherence and PLV that is more robust against inflation due to finite numbers of trials for coherence [53] or time points for PLV. The trial correction formula was mainly relevant for the coherence estimates. For clarity, the number of phase estimates was represented by the number of trials N for coherence and the number of time points T for PLV. The unbiased estimator of squared PLV is the following [58]:

$${\text{PLV}}^{2}=\frac{1}{T-1}\left(({\text{PLV}}^{2}T\right)-1)$$ (19)

where *T* is the number of sample points. The same was done for deriving the expected PLV (i.e., the PLV obtained in the absence of any noise). For coherence the formula is:

$$Coh^{2}=\frac{1}{N-1}\left(\;\left(Coh^{2}N\right)-1\right)$$ (20)

where N is the number of trials.

### The Transfer Entropy (TE) measure

In addition to the phase-locking estimation, we also quantified the directed information flow between network X and Y by applying (delayed) transfer entropy measure TE according to [43]. TE measures a directed flow of information between two processes. The TE measure allowed us to more concretely demonstrate the relevance of PLV and coherence for information transmission among neural networks. For a given spike train from neuron I, TE gives the amount of reduced uncertainty when using knowledge of preceding time bins in the spike train from neuron J over using knowledge of preceding time bins in the spike train from neuron I itself. We quantified the directed information flow from RS neurons of network X to RS neurons of network Y. For quantifying TE, we selected 80 RS neurons of each network defining 80x80 combinations. We concatenated the spike trains of all trials to increase sensitivity. Then TE was applied for all 80x80 combinations, where the transfer entropy for different delay time values (delays up to 16ms were included) was computed and then combined. The TE was averaged overall 80x80 combinations. The TE of spike train from neuron I (binary 0/1 vector where 1 denotes spike) on the spike train from neuron J was computed as follows [43]:

$$TE_{I\to J}\left(d\right)=\sum \;p(i_{t+1},i_{t}\;,\;j_{t+1-d})log_{2}\frac{p\;(i_{t+1}|i_{t}\;,\;j_{t+1-d}\;)}{p(i_{t+1}|i_{t}\;)}$$ (21)

where d is the delay (here in terms of 1ms time steps),i_t_ or j_t_ is the status of spike train at time t (0 = no spike, 1 = spike) of neuron I or J respectively.

## Results

In the following, we will first show simulations of coupled phase-oscillators with no intrinsic phase noise. We used the simpler case first as it allowed us to analyze the generative model analytically, in addition to numerical simulations to precisely understand the behavior of spectral coherence. In a next step, we added intrinsic phase noise to make the phase evolution more similar to experimental data and we then also compared the behavior of spectral coherence to the PLV method. As a last step, we extended the results from the phase-oscillator simulations to two mutually interacting gamma-generating spiking neural networks representing a biophysical plausible model of gamma oscillations.

### An evaluation of spectral coherence as a measure of phase locking between two intrinsically noise-free coupled phase-oscillators as a function of added extrinsic noise

We systematically investigated the behavior of coherence, for different SNRs, as a function of detuning Δω (PrFM) and different levels of PrAM without intrinsic phase noise. To that aim, we compared in our simulations the analytically derived expected PL^2^ of two interacting phase-oscillators with the analytically derived spectral coh^2^ as well as the numerically estimated coh^2^.

**Fig 2:**
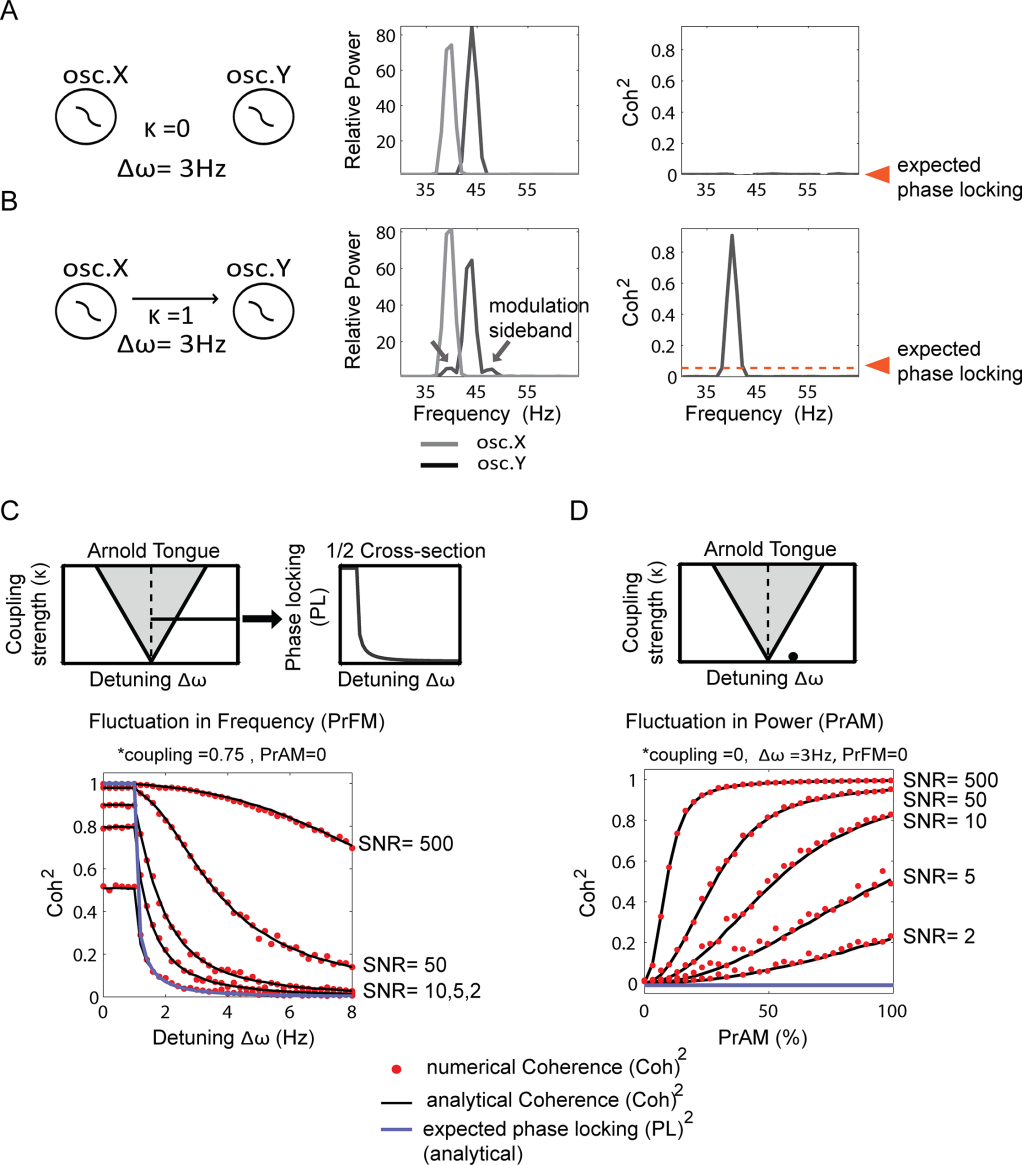
Analytical and numerical results of Coherence estimation of phase-locking with different levels of extrinsic (measurement) noise. In (A-B) we first show two examples. In (A) oscillator X and Y had a detuning of 3Hz and did not interact. The power spectra (middle panel) show the two power peak of the two oscillators. The coherence spectrum (right panel) was flat as expected. In (B), the oscillators did interact (κ = 1), where oscillator X influenced the phase trajectory of oscillator Y. The power spectra of oscillator Y show two extra power peaks with ± the detuning. These are the so-called modulation sidebands, well described in the cross-frequency coupling literature [62]. Notice that one of the sidebands overlap with the power peak of oscillator X. The coherence spectrum show a strong phase-locking estimate, much higher than expected. This is because the coherence estimate reflected mostly the locking between the sideband of oscillator Y and the main power peak of oscillator X, which can be completely unrelated to the actual phase-locking of oscillators X ad Y. (C) Rendering of the Arnold tongue, shown with a 1/2 cross-section at the level of a 0.75 coupling strength, for which phase locking values are plotted as a function of positive, increasing intrinsic frequency differences between oscillators X and Y (Δω). Here, we did not add PrAM to the oscillatory signal. We compared the numerically (red dot) and analytically derived (black line) Coherence with the analytically derived true phase-locking (purple line) between two oscillators as a function of frequency detuning (Δω) and different levels of SNR. We used trial-number corrected squared coh values to minimize inflation due to a finite number of trials. In the partially synchronized states associated with different Δω values in the selected coupling condition, we observed strong deviations of Coherence from the true locking. The coh^2^values became more inflated with higher SNR. The numerically computed coh^2^ matched with the analytically derived coh^2^. (D) The impact of different levels of PrAM is shown with different level of SNR. The oscillators were uncoupled and hence asynchronous (in the condition indicated by the fat dot at the bottom of the Arnold tongue) and the true locking was therefore 0. The oscillators had a phase precession of 3Hz (chosen condition is located off the midline of the Arnold tongue). We observed strong deviations from the true locking with increasing PrAM and SNR. The numerically and analytically derived values matched.

We first show two illustrative example conditions. In the first example (Fig 2A), the phase-oscillators were uncoupled and the oscillators X and Y had a detuning Δω of 3Hz. In the middle panel of Fig 2A, the Fourier power spectra can be observed with two power peaks; one for each oscillator. The right hand panel in Fig 2A shows the absence of a coherence peak as expected. In the second example (Fig 2B), oscillator X interacted unidirectionally with oscillator Y with κ = 1. Strikingly, in the power spectrum of oscillator Y, two side peaks can be observed around the central power peak with a distance of ±Δω = 3Hz. These are so-called modulation sidebands, which have been described in the cross-frequency coupling (CFC) literature [59–61]. They arise because the synchronization force induced by oscillator X on Y (see interaction term) leads to systematic phase-relation dependent frequency modulations (PrFM) at the 3Hz detuning frequency Δω. Notice that the left modulation sideband power peak of oscillator Y and the power peak for oscillator X are at the same frequency. To the right, a strong coherence peak can be observed which is much higher than the expected phase-locking value. The coherence peak is at the frequency where oscillator X and the left modulation sideband of oscillator Y have their power peak. The coherence peak hence reflects the phase-locking between the PrFM of oscillator Y (induced by oscillator X) with oscillator X itself.

We then systematically compared the numerical and the analytically derived coh^2^ (Fig 2C) with the true phase-locking, for different SNR of extrinsic noise, as a function of detuning frequency *Δω*. The SNR was manipulated by changing the level of extrinsic noise. The oscillator X was unidirectionally coupled to oscillator Y by *κ = 0*.*75* (horizontal line in left upper panel Fig 2C). Oscillator X had a frequency of 40Hz. At the constant coupling strength, the frequency of oscillator Y was shifted from 40 to 48Hz in step size of 0.25Hz (detuning *Δω* 0 to 8Hz; schematically shown in top right panel Fig 2C). The values chosen corresponded to a half cross-section of the (1:1) Arnold tongue. In the above-defined conditions, we evaluated SNRs of 500, 50, 10, 5 and 2 on coherence (Fig 2C bottom panel). The numerically and analytically derived coh^2^ (red symbols, black line in Fig 2C bottom panel) matched well for all conditions, and we therefore do not distinguish them further. For low detuning Δω (inside the Arnold tongue) and high SNR, the oscillators were in full synchrony and showed a coh^2^ value of 1, matching the true phase locking (blue line). For lower SNR the noise started to affect the coh^2^ estimate more substantially. As white noise was uncorrelated between oscillators, the white noise tended to decrease the coh^2^. At an SNR of 2, the coh^2^ gave an estimate reduced by 50%. At a particular detuning frequency (*Δω* = 1.5Hz, around the edge of the Arnold tongue) the phase-locking between the oscillators started to drop (partially synchronized state). The oscillators were therefore not completely frequency-frequency locked and had disparate frequencies. In this case, the coh^2^ (red symbols in Fig 2C bottom panel) deviated strongly from the PL^2^ indicated in the blue line in Fig 2C (bottom panel), and depended strongly on the SNR. Without any additive white noise (high SNR), the coh^2^ estimates were 1 or approached 1 despite the PL^2^ being very low. The lower the SNR, the more noise affected the estimate and the more coh^2^ turned towards 0. Importantly, with higher SNR, the estimates converged not on the true phase-locking (with the exception of perfect locking), but towards a phase-locking of 1. Nevertheless, the coh^2^ estimates reflected to a certain degree the underlying changes in the PL^2^ as a function of detuning if noise was present. This is because the amplitude of the modulation sideband (induced by PrFM) is a function of the PL (see below). The noise unmasks this dependence. This is because the amplitude of the modulation sideband decreases with detuning, the SNR decreases as well if noise is present. Because coherence is sensitive to SNR, the value will reflect the SNR changes of the modulation sideband peak. Notice that at very low SNR the values (<2) coh^2^ converged to 0 as the noise was uncorrelated between oscillators.

We then investigated the dependence of coh^2^ estimates on the presence of PrAM. For simplicity, we used conditions in which the phase-oscillators were uncoupled (black dot Fig 2D upper panel) and hence the true phase-locking was 0 for all conditions (blue line bottom panel Fig 2D). The oscillators had a detuning of 3Hz. We evaluated different levels of PrAM ranging from 0 to 100%. We used the same SNR conditions as before. Again, the numerically and analytically derived coh^2^ estimates matched well. We observed that coh^2^ estimates deviated from the true phase-locking as a function of both the level of PrAM and the level of SNR (Fig 2B). The higher the PrAM was set, the higher the coh^2^ values became, and hence the more coh^2^ deviated from true locking. This was because higher PrAM leads to higher amplitude of the modulation sideband making it more dominant above to the noise. Therefore, similar to the case with PrFM, with increasing SNR, the coh^2^ estimates did not converge towards the expected phase-locking of 0, but to a phase-locking of 1. These results show that spectral coherence is not a pure phase-locking measure, but also reflects phase-relation dependent amplitude fluctuations. Note that only one oscillator had amplitude fluctuations, hence the amplitude correlation between oscillators was 0.

#### Conceptual and mathematical understanding of the underlying cause of spectral coherence deviations from expected phase-locking

Oscillatory processes that exhibit phase synchronization without complete phase locking, are in a partially synchronized state, and will show characteristic PrFM and likely also PrAM during phase precession. These systematic modulations of the oscillation frequency and amplitude occur at the frequency of the phase precession (equal to the frequency difference between oscillators). These types of modulations can be seen as a form of cross-frequency coupling ‘CFC’ [62], here between oscillators of nearby frequencies and with the phase-relation as the modulation variable. The CFC between two oscillations of nearby frequencies (e.g. 42Hz and 45Hz) leads to so-called modulation sidebands (SM) that are located ±3Hz nearby the main power peak. These ‘modulation sidebands’ between lower and higher-frequency oscillations have been previously described in CFC literature, with specific reports on the modulation of the amplitude of the higher frequency oscillations by the phase of the lower frequency oscillation [59–61]. We will describe below in more detail the underlying causes of SM.

Here, we were interested in computing the CFC phase-phase locking between the two oscillatory signals [63]. To that aim, one would need to compute the relationship of the phases at the higher frequency (45Hz) to the phases at the lower frequency (42Hz). Spectral coherence was not being expected to be applicable here because the oscillators X and Y did not share power at common frequencies. Yet, when applied, spectral coherence measures yielded inflated values. This was because one of the modulation sidebands (by definition) overlapped with the power peak of other oscillators. Note that this coherence estimate is bound to be incorrect, because it computes coherence frequency per frequency, yet here, one would need to compute coherence across frequencies. Notice also that in practice, it might be difficult to discover these modulation sidebands in largely overlapping power spectra in experimental data due to intrinsic and extrinsic noise, yet they might still affect the computations of coherence. In fact, as we show below, this type of CFC interactions occur even if power spectra completely overlap (zero mean difference), yet there is frequency variation from trial to trial.

Below, we explain in more detail why the modulation sidebands are induced by PrFM and PrAM and how phase-locking computed with coherence leads to erroneous estimates of phase locking under these conditions. To understand why spectral coherence gave incorrect estimates, one needs to understand how these modulation sidebands arose and how they affected the coherence estimates. Oscillatory synchronization can lead to systematic PrFM and PrAM which both can induce modulation sidebands. In the Methods A in S1 File we show mathematically how for the noiseless case the modulation sideband SM is related to PrFM and PrAM. It can be shown that the amount of modulation sideband induced by PrFM (if PrAM is also absent) is a function of the (expected) phase locking scaled by the oscillation amplitude.

$${\text{SM}}_{\text{PrFM}}=A\;\text{PL}$$ (22)

Hence, for a given oscillation amplitude, the amount of SM was proportionally related to PL. For SM induced by PrAM (without PrFM, PL = 0) it can be shown that it is a direct function of PrAM modulation strength (α) scaled by the oscillation amplitude.

$${\text{SM}}_{\text{PrAM}}=A\frac{\alpha }{2},\;\text{if}\;\text{PL}=0$$ (23)

The SM induced by either PrFM or PrAM in oscillator Y has a constant phase-relation to the oscillator X inducing the PrAM or PrFM. Hence, in the noiseless case the coherence peak between the modulation sideband SM of oscillator Y and the oscillatory X has to be 1 (Methods A in S1 File). So whatever detuning one chooses or even in the case of no coupling, the coherence peak will be 1 (if ω_x_ ≠ ω_y_). Adding extrinsic noise to the signals, being uncorrelated between the oscillators, had the overall effect of decreasing the coherence estimates. Increasing the extrinsic noise level caused the coherence estimates to converge towards 0.

However, the effect of adding noise to the signal had more implications than decreasing the coh^2^ values towards 0. Adding extrinsic noise made the coh^2^ related to the true underlying phase-locking changes with detuning. By ‘related’ we mean that the coh^2^ and the (expected) PL^2^ values were correlated, yet not implying that the values matched exactly. In Fig 2C, for different SNRs the coh^2^ deviated substantially from the PL^2^ values. Yet, they shared the property that they overall decreased with detuning. One could raise the question of how this is possible given that the coherence peak reflected the phase consistency between the modulation sideband of oscillation Y and oscillation X, while this phase consistency was defined as perfect for all coupling and detuning conditions. The reason for this lies in the property of SM_PrFM_ to change its amplitude as a function of PL (see Eq 22). The effect of including extrinsic noise can now be understood as follows: In the simulations (Fig 2C), when the detuning decreased, the PL increased. Because the PL increased, the amplitude of SM_PrFM_ increased, and given a specific fixed noise level the ratio of SM_PrFM_ amplitude versus noise amplitude also increased, which meant that the SM_PrFM_ SNR increased. Because the SM_PrFM_ SNR increased, the coh^2^ values increased. Through this relationship, for a large range of extrinsic noise levels, there was a rough relation between the PL and the coherence estimates. Note that this is only the case for SM_PrFM_, because SM_PrAM_ is unrelated to the underlying PL (see Eq 23).

#### Deriving analytically the spectral coherence estimates

Adding uncorrelated white noise (simulated ‘measurement error’) decreased the coherence values in general. The amount of reduction was a function of the ratio between the signal power and the noise power (the SNR). The essential ratio determining the spectral coherence peak is the ratio between the amplitude of the modulation sideband and the white noise amplitude. There are four variables affecting the true phase difference between the two oscillators: the amplitudes of two independent white noise processes and their phase values. The power from a white noise process is known to have a chi-square distribution of order 2 [64] with mean power being equal to its variance. For amplitudes it corresponds to a chi-distribution of order 2, for which the probability density function is given by:

$$\chi \left(A;k\right)=\frac{2^{1-k/2}A^{k-1}e^{-\frac{A^{2}}{2}}}{\Gamma {\left(\frac{k}{2}\right)}_{}}$$ (24)

$$\chi \left(A;2\right)=Ae^{-\frac{A^{2}}{2}}$$ (25)

The phase distribution of a white noise process is the uniform distribution:

$$\varphi_{W}\left(t\right)=\{\begin{array}{l} \frac{1}{2\pi },\;-\pi \leq t\leq \pi \\ \;0,\;otherwise \end{array}$$ (26)

The actual phase of each oscillator is the complex vector addition of the signal and noise. The product of these complex values between the two oscillators gives the actual phase difference. The spectral coherence evaluated at the frequency where the oscillation *X* shares power with the modulation sideband of oscillation *Y* is:

$$Coh=\left|\int_{A_{X}=0}^{A_{X}=\infty }\int_{A_{Y}=0}^{A_{Y}=\infty }\int_{\varphi_{Xw}=-\pi }^{\varphi_{Xw}=+\pi }\int_{\varphi_{Yw}=-\pi }^{\varphi_{Yw}=+\pi }\chi \left(A_{X},2\right)\chi \left(A_{Y},2\right)\varphi_{W}^{2}e^{\text{iArg}\left(\left({\text{A}}_{\text{X}}{\text{e}}^{{\text{i}\varphi }_{\text{Xw}}}{+\text{S}}_{\text{X}}{\text{e}}^{{\text{i}\varphi }_{\text{X}}}\right)\left({\text{A}}_{\text{Y}}{\text{e}}^{\text{i}\theta \varphi }{+\text{S}}_{\text{Y}}{\text{e}}^{\text{i}\theta \varphi }\right)\right)}d\varphi_{Xw}d\varphi_{Yw}dA_{X}dA_{Y}\right|$$ (27)

where *A*_*X*_ and *A*_*Y*_ represent the amplitudes of the white noise. φ_*Xw*_ and φ_*Yw*_ represent the phases of the white noise of the oscillator *X* and *Y* respectively. *S*_*X*_ is the amplitude of oscillator *X* and *S*_*Y*_ is the amplitude of the modulation sideband of *Y*. We assumed for simplicity that the characteristics of PrAM and PrFM are constant therefore the oscillator *X* and the sideband modulation of oscillator *Y* had a constant phase relationship (ϕ_X_—ϕ_y_ = constant). The accuracy of the estimates can be seen in Fig 2C and 2D. They fitted the numerically estimated coherence estimate well, demonstrating that the induction of SM by PrAM and PrFM as well as the SNR were the underlying determinants of the peaks in the coherence spectra.

### Comparing coherence with PLV as measures of phase locking between two coupled intrinsically noisy phase-oscillators as a function of added extrinsic noise

The previous sections have demonstrated severe limitations for coherence as a measure of phase locking. In various studies [28,34,35,65] it has been proposed that phase locking approaches like the phase-locking value (PLV)—which are based on the reconstruction of the instantaneous phase, here by applying Hilbert transform (HT) and singular spectrum decomposition (SSD)—can be a viable alternative to coherence. To make a fair comparison between coherence and PLV, it is necessary to do this on model data that more accurately reflect data as they could be measured empirically. We achieved this by making the phase oscillators intrinsically noisy. In previous sections this was not done, as the main aim in preceding sections was to illustrate maximally the conceptual and mathematical problems of using coherence as a measure of phase relations between oscillators. Here, the aim will be to use model data that show noisy characteristics closer to those that could be recorded empirically (e.g., in electrophysiological recordings), but still with the underlying phase relations known, which are to be estimated by coherence and PLV. In addition to including intrinsic noise, we added different levels of extrinsic (measurement) noise as in previous simulations to manipulate SNR.

Intrinsic noise was modeled as a noise process Np(t) (pink, scaling factor of 1, SD = ~1.5Hz) added in the phase-oscillator equation affecting the phase evolution of the oscillator. The properties of the intrinsic noise process did not change over the simulations conditions. It is termed ‘noise’ as the phase variability is of unknown origins. Notice that including intrinsic noise affects the phase-evolution, but this variation is of biological interest and needs to be included for phase-locking estimation. This contrasts with extrinsic (measurement) noise, which is unrelated to the dynamics of interest and should be ignored. What is the effect of using phase oscillators that are intrinsically noisy? Without noise and thus with a fixed intrinsic frequency, oscillations were very narrow-banded and the frequency distributions of the two coupled oscillations in many cases non-overlapping. Under these conditions, coherence was shown to be a poor estimator of phase relations. Measured neural oscillations however (e.g. gamma-band) have broader spectral power peaks [14,30,66,67]. This indicates that neural oscillations exhibit rapid phase and frequency dynamics, which can be expected from noisy and complex networks [68] of which brain networks are prime examples. Sources of the variability in oscillation frequencies and relative phases include intrinsic noise/instability within a network [66], perturbations from other networks [17] and cross-frequency interactions [69,70].

**Fig 3:**
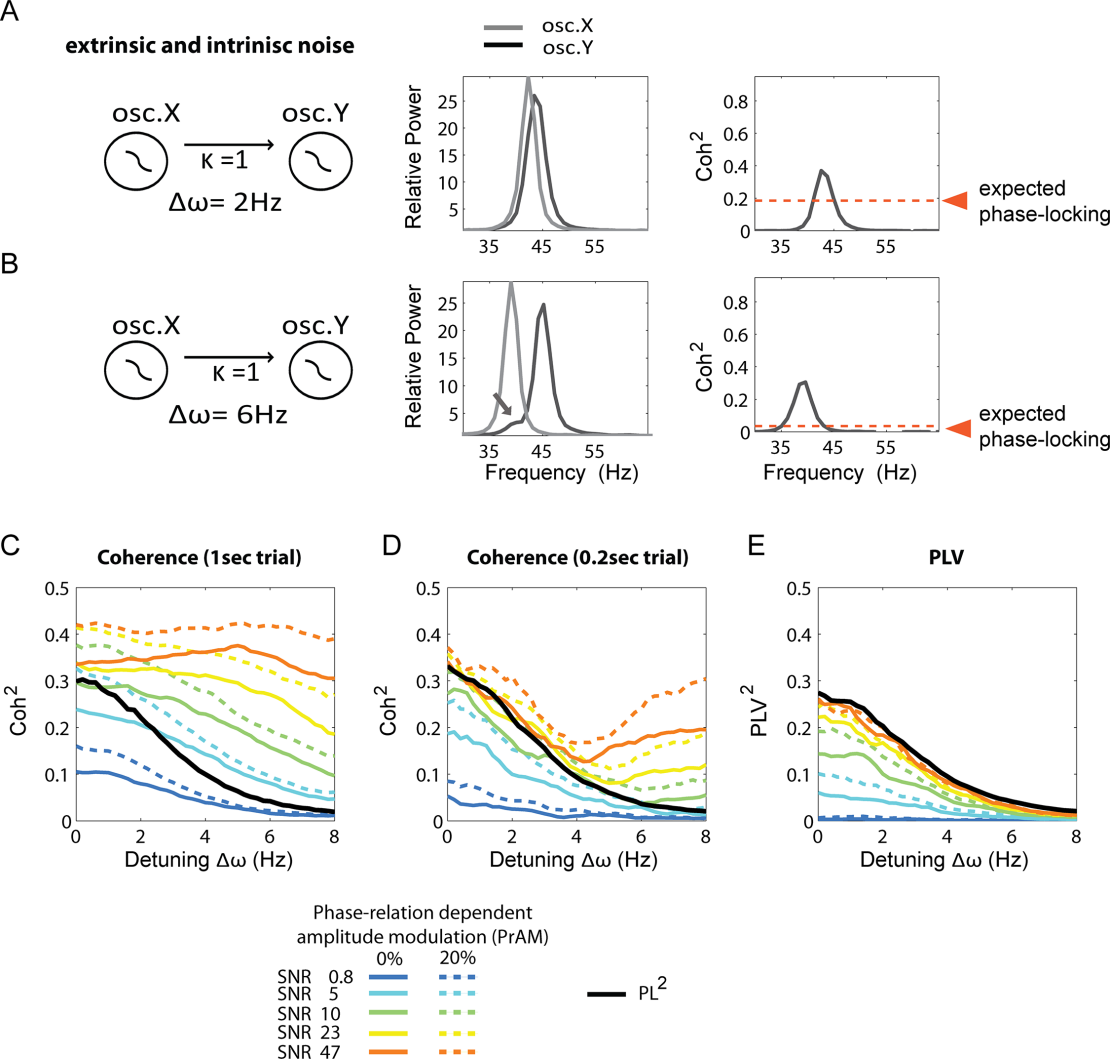
Comparison of spectrally and non-spectrally based approaches for the estimation of phase locking. The figure shows numerical results of Coherence and PLV estimates of phase-locking between phase-oscillators with both dynamical noise (more broadband) and different level of extrinsic (measurement) noise (uncorrelated between oscillators). First two examples are shown. (A) Oscillators X and Y are interacting (κ = 1 X-> Y) with a detuning of 2Hz (X = 38Hz, Y = 40Hz). The power spectra (here in the gamma range, although exact range is irrelevant here) are shown in the middle-panel. The power spectra do largely overlap and the modulation sidebands cannot be easily observed (but are present in the data). The coherence spectrum (right panel) gave a phase-locking estimate larger than expected with a peak at 38Hz (where the left modulation sideband of oscillator Y overlaps with the power peak of oscillator X). In (B) the detuning was increased to 6Hz (X = 34Hz, Y = 40Hz) with same coupling conditions. Now the left modulation sideband of oscillator Y can be observed as a small peak at 34Hz. The coherence spectrum gave a phase-locking estimate much larger than expected reflecting the influence of the modulation sideband. (C) A 1/2 cross-section of the Arnold tongue, similar to Fig 3A, is shown. The continuous lines represent simulations without PrAM and the dashed lines represent simulation with a PrAM of 20%. We compared the coherence to the expected phase-locking. We used the noise-free instantaneous phases of the phase-oscillators to compute the PL^2^, which was a good estimator of the analytically derived true phase-locking. We observed that the coh^2^ values deviated strongly from the expected phase-locking. The exact deviation depended on the detuning frequency and SNR. Including a PrAM of 20% led to a further inflation of the coh^2^ values. Note also the deviations of coh^2^ from the PL^2^ at a zero detuning frequency. (D) The same analysis as in (C) but with PLV values estimated by the SSD-HT method. We observed that for higher SNR the estimate behaved better and remained close to the expected phase locking. At lower SNR the PLV^2^ showed lower than expected values due to the effect of (uncorrelated) noise. Including a PrAM of 20% led to an inflation of PLV^2^ values in the lower SNR only.

In Fig 3, we show data where oscillator X was unidirectionally coupled with oscillator Y (κ = 1, X→Y). The pink noise (applied to X and Y) was uncorrelated between the oscillators. Including the pink noise term had two effects: It broadened the range of frequencies of the oscillator. Second, because the intrinsic frequency varied due to noise, the oscillators did not have a precise position on the detuning dimension anymore, but it varied over time. That makes full synchrony very difficult to achieve, because strong noise fluctuations kick the oscillators out of their ‘attractor’ phase-relation [7]. Even if the mean detuning is Δω = 0, the phase-locking strength might be lower than 1 due to the intrinsic phase noise. In the simulations described in Fig 3 a systematic comparison was made between the ability of coherence to estimate expected phase locking and the ability PLV to estimate true phase locking, for 5 different SNR (0.8, 5, 10, 23 and 47). Note that SNR refers here to extrinsic (measurement) noise that is uncorrelated between oscillators. Further, we evaluated conditions without any PrAM and conditions with 20% PrAM. Simulated data comprised for each condition 500 trials of 3s length. The first 2s were discarded of each trial to assure that the oscillators reached stable state. For coherence, we analyzed data using the full 1s window or by splitting the 1s window into 0.2s windows. For these simulations, we did not derive the analytical phase-locking between the two oscillators, but relied on a numerical estimation only. For the same conditions as described above, we then computed PLV and compared with the expected phase locking PL (i.e. the known phase locking prior to the addition of extrinsic noise sources). Below we describe the simulation results in detail.

Two examples are shown illustrating the behavior of coherence under two conditions of detuning. In Fig 3A (left panel) oscillator X and Y (κ = 1, X→Y) had a detuning of Δω = 2Hz. Their power spectra (middle panel) overlapped largely. Note that experimentally reported relative power values can range from ≥1 to ≥25 (or higher), depending on the oscillation band, the recording method used and neuronal structure investigated. Hence, the (high SNR) power values simulated here are in line with experimentally possible values. Note furthermore that the modulation sidebands are basically invisible. However, they are still present (e.g. they can be observed in single-trial power spectra) and affect considerably the resulting coherence spectra as depicted to the right in Fig 3A as the coh^2^ estimate (peak height) was higher than expected (right hand panel). In Fig 3B we used the same configuration, but now with a detuning of Δω = 6Hz. Notice that now, the left modulation sideband of oscillator Y, induced by oscillator X can be seen (indicated by arrow) as a small peak coinciding with the peak power frequency of oscillator X. Also in this case, the peak of the coherence spectrum was much larger than expected.

In Fig 3C, we systematically modulated the detuning Δω to observe the behavior of spectral coherence estimates. The PL^2^ was at ~0.3 for a mean 0Hz detuning (Fig 3C). Despite the fact that the mean frequency of the oscillators matched, considerable instantaneous frequency variation was present centered around 0. Due to the phase noise the oscillators could not reach full synchrony, and instead exhibited phase precession. We then compared coh^2^ estimates computed for a 1sec trial length to the PL^2^ values. The coh^2^ estimates were dependent on the SNR. With higher SNR, coh^2^ did not converge to the PL^2^, but exceeded it. Including a 20% PrAM led to a further inflation of the coh^2^ estimates. Hence, despite the oscillators having a matching mean frequency, coh^2^ estimates showed deviations (Fig 3C, left panel) from the expected value. Increasing the detuning frequency Δω led to a smooth decrease of the expected phase-locking. The coh^2^ estimate of the highest SNR did not decrease at all for a large range of detuning values. Moderate SNR levels (23, 10, 5) showed a very slow decrease of coh^2^ as function of detuning, but still deviated from the PL^2^. At low SNRs the effect of the extrinsic white noise became dominant and coh^2^ estimates were converging to 0 (as the noise was uncorrelated between oscillators). Applying the 20% PrAM led to an overall inflation of the coh^2^ estimates. Note that the inflation by PrAM increased with increasing SNR (as expected from Fig 3C). These results show that also under more realistic oscillatory dynamics, spectral coherence exhibits strong deviation from the PL, thus confirming the analysis of the previous simulations without intrinsic phase noise.

We re-analyzed the same simulation data, but restricted the coherence estimation to 0.2sec trials length (Fig 3D) by using the time window 2–2.2sec after simulation onset (the first 2 seconds were discarded). We explored the 0.2sec time-window coherence, because spectral coherence is also applied in the context of TFR, e.g. using short-time Fourier transform. We used 0.2sec time-windows also because it is a typical time-scale used for the TFR analysis of higher frequency oscillations in neuroscience [8,71]. For lower oscillations longer windows are usually used. The use of a 0.2sec time-window restricted the frequency resolution to 5Hz. This had an impact on the coherence estimation properties. For detuning conditions below 5Hz, the coh^2^ estimates converged to the expected phase-locking with higher SNR. Starting from detuning ~4Hz the coh^2^ estimates lost this property and deviated again as shown in Fig 3C, obtained with 1sec trial lengths. Including 20% PrAM affected the coh^2^ estimates, yet much less below 4Hz than above 4Hz detuning (Fig 3D), and also much less compared to 1sec trial length coherence (Fig 3C). The particular detuning value at which the coh^2^ estimates started to lose the property of converging towards the PL with increasing SNR, was a function of the time-window length and its associated frequency resolution. This is because the coherence deviation from the PL depends on the separation of the oscillation band and the modulation sideband. If, due to short time-window length, the frequency resolution is low and spectral leakage is large, the phase estimates of the cross-spectral density will largely reflect the oscillation band (and not the modulation sideband). This is because the oscillation band has larger amplitude than the modulation sideband.

Comparing Fig 3C and 3D, one could argue that decreasing the time-window for coherence estimation to 0.1sec or lower (generally problematic for lower frequency oscillations) could solve the issue of coherence misestimations induced by modulation sidebands. Using very small time-window for coherence estimation can be seen as approximating instantaneous phase, however with the cost of low frequency resolved coherence spectra. It can be questioned whether spectral coherence was designed for this, and whether it is not better to use methods that were specifically designed for instantaneous phase reconstruction. A further drawback of very short-time window coherence is that, if the signal consists of several components (for example any combination of theta, alpha, beta or gamma components), the coherence estimates might be affected by component mixing due to the very low frequency resolution. In comparison, SSD helps to avoid this problem in a completely data-driven manner [36].

In Fig 3E, we show the PLV^2^ estimates based on the same testing dataset as used in Fig 3C. In brief, for the PLV approach, the signals were first decomposed using SSD [36,56]. We chose the SSD oscillatory component with the most power in the expected frequency range. The SSD helped to reduce impact of extrinsic noise on the instantaneous phase estimation and further assured that the signals were mono-component. We applied the Hilbert transform to the extracted components, to extract the instantaneous phases. From the instantaneous phase-relation we then computed PLV^2^. We observed that for increasing SNR the PLV^2^ estimates converged to the PL^2^ over the whole detuning frequency range (0 to 8Hz). For lower SNRs, the PLV^2^ estimates were lower than the PL^2^, because the extrinsic noise started to affect the estimates more substantially. At the lowest SNR, the PLV^2^ estimates were very close to 0. The addition of a 20% PrAM affected the lower SNR condition, but not the higher SNR conditions. Because the amplitude (or power) fluctuates with phase-relation, the SNR fluctuates with phase-relation, as the SNR is the ratio of signal amplitude and the (extrinsic) noise level. This leads to an SNR-weighted phase-relation distribution causing the PLV estimate to increase. For higher SNR this effect vanished. Overall, the PLV^2^ estimates behaved much more appropriately in the conditions tested compared to coh^2^ estimates, particularly in the higher SNR ranges. A drawback of PLV (which is the same for all methods reconstructing instantaneous phase) is that it is less robust against extrinsic noise (e.g. compare blue lines of Fig 3E with Fig 3C).

### Comparing coherence with PLV during manipulations of input drive and connectivity between two coupled gamma-generating excitatory-inhibitory neural networks

**Fig 4:**
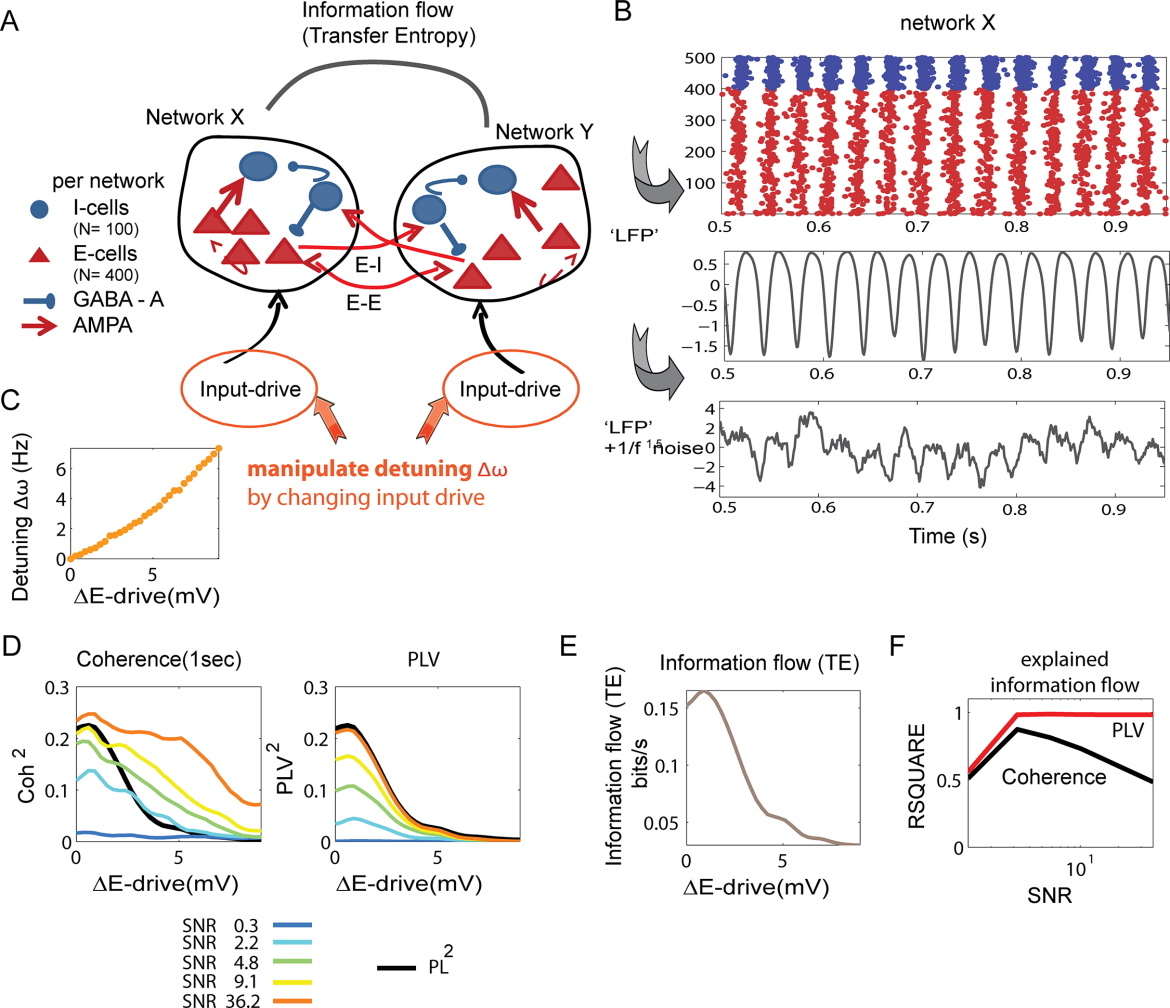
Testing performance of coherence and PLV in the estimation of phase-locking performance and information flow of two interacting gamma-generating spiking networks receiving different detuning levels. (A) The network architectures. Two interconnected excitatory-inhibitory networks consisting of 100 inhibitory cells (fast-spiking type) and 400 excitatory cells (regular spiking) were simulated using Izhikevich formalism [39]. Neurons were interconnected with AMPA (excitatory) and GABAA (inhibitory) connections. The networks generated so called ‘pyramidal-interneuron network gamma’ (PING). The two networks where weakly interconnected by E-I and EE interconnections. The detuning was manipulated by altering the difference in excitatory input drive (to E-cells) between the networks. We used the experimentally and theoretically established observation that the frequency of gamma oscillations is tightly linked to input drive. (B) Generation of test-signals. From each of the 300 1sec trials the ‘LFP’ (population signal) was extracted from each network by summing and smoothing (pseudo Gaussian function of 3ms width) the E-cell spikes. Then we added 1/f noise (exponent = 1.5) to manipulate SNR. Compared to the phase-oscillator model, phase-relation dependent amplitude modulations (PrAM) were generated intrinsically in the model. (C) By changing the relative excitatory input drive to E-cells between the networks, we could manipulate the detuning (frequency difference). (D) Coh^2^ (in the gamma frequency range 30-50Hz) and PLV^2^ as a function of input drive difference (ΔE-drive) between networks. Different line colors represent difference SNR (relative power). The black line represents the PLV^2^ with no noise added (PL). (E) Information flow (combined directions), as measured by transfer entropy (TE), as a function of input drive difference (ΔE-drive) between networks. (F) Variance in information flow explained by coh^2^ (black) and PLV^2^ (red) as a function of SNR derived by computing pearson correlation.

So far, we have used a rather abstract model of oscillatory phase synchronization. Although it has been shown that the phase-oscillator equations and the theory of weakly coupled oscillators (TWCO) constitute a fruitful and powerful framework to describe and understand oscillatory synchronization in biophysical systems like neural networks [7,37,45–48,52,72–77], we will now show that the same principles described in the data generated by two coupled phase-oscillators, hold true in more complex datasets generated by two interacting excitatory-inhibitory spiking neural networks [40,52,72,73]. The networks used here generated gamma oscillations of the so-called ‘pyramidal-interneuron gamma’ PING type [39]. The overall structure of the network is shown in Fig 4A and the derivation of the network oscillatory signal is described in Fig 4B (see also Methods). In short, each network consisted of excitatory (E-cells) and inhibitory cells (I-cells) which interacted through synaptic AMPA (excitatory) and GABA-A (inhibitory) interactions [40]. The two networks interacted through weak AMPA connections targeting E-cells as well as I-cells of the other network. For each network we derived a population oscillatory signal which was computed as the smoothed combined spiking of all E-cells. For changing the noise-levels we added different amount of 1/f^1.5^ noise to the population signals.

As with the phase-oscillator generated data, we applied in the same manner spectral coherence and PLV. In addition to the phase-locking estimation, we also quantified the directed information flow between network X and Y by applying transfer entropy measure TE according to [43]. TE measures a directed flow of information between two processes. We quantified the directed information flow from E-cells of network X to the E-cells of network Y (and vice versa). The TE measure allowed us to more concretely demonstrate the implications of coherence misestimations for the understanding of information transmission among neural networks.

**Fig 5:**
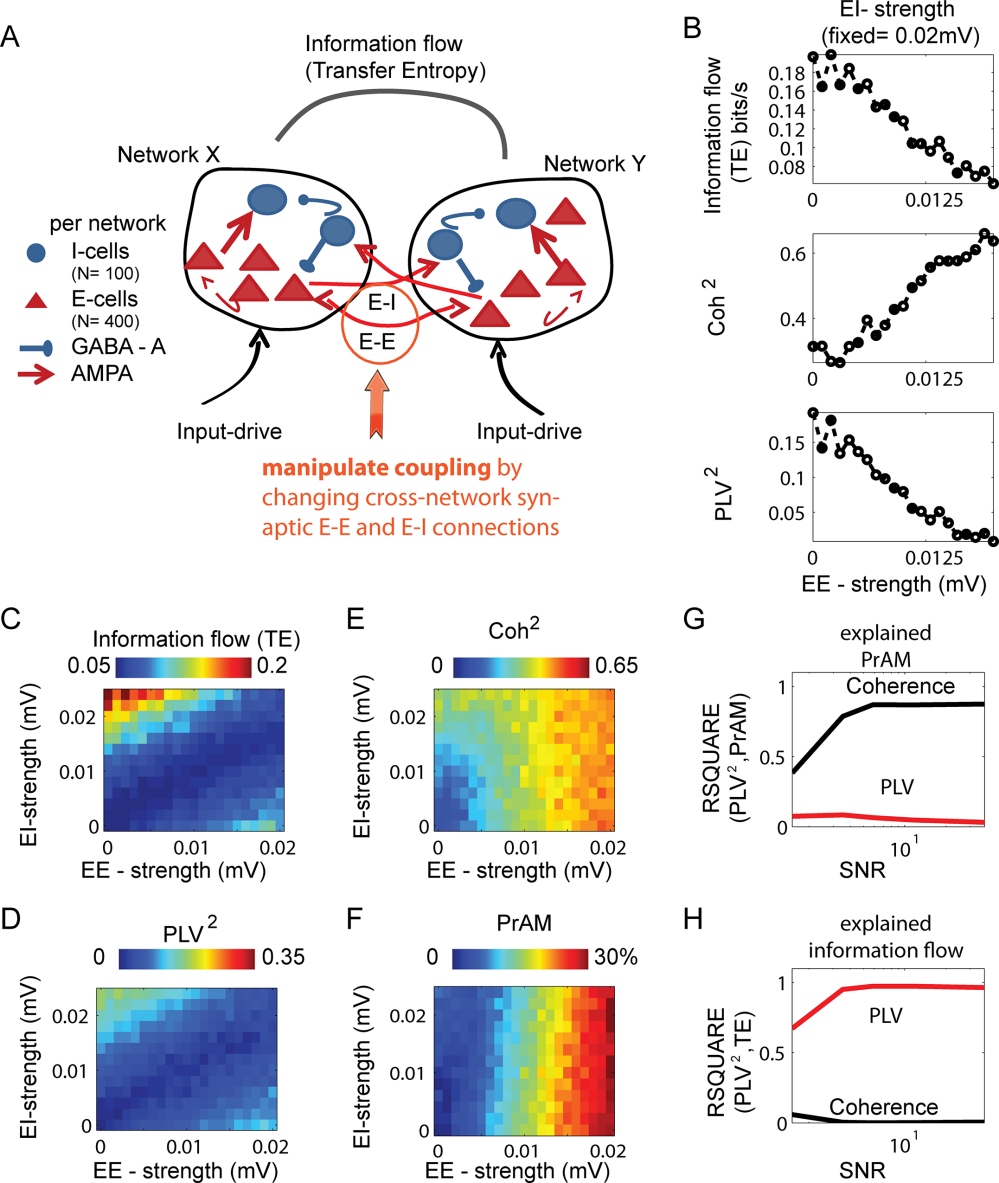
Testing performance of coherence and PLV in estimation of phase-locking performance and information flow of two interacting gamma-generating spiking networks among which the coupling is manipulated by changing the strength of cross-network synaptic connections. (A) The network architecture as shown in Fig 4. Here, the coupling strength κ between the networks was manipulated changing the E→I and E→E values. The input drive difference was kept the same (Δ1.5mV). B) Example (SNR = ~10) of EE- strength manipulation (0 to 0.02mV) with a fixed IE strength of 0.02mV. The top-plot shows the information flow as measured by transfer entropy (TE), combined for both directions as a function of EE-strength. Middle- and lower panels show the same for coherence^2^ and PLV estimate respectively. For this particular combination the coh^2^ estimate behaved oppositely to PLV^2^ and information flow. (C-F) Surface plots representing effects of all combinations of E→I and E→E strengths on information flow (C), coh^2^ (D), PLV^2^ (E) and PrAM strength (F). A comparison of surface plots reveals that coherence not only reflects changes in PLV or information flow with coupling manipulations, but also changes in PrAM. (G-H) Variance in coh^2^ and PLV^2^ explained by PrAM (G) and by information flow (TE) (H) plotted as a function of SNR. Changes in coh^2^ values predominantly reflected changes in PrAM (G), yet explained little variance in information flow between networks with coupling manipulations (H). The opposite was true for PLV^2^.

Below we will show results from network simulations where we manipulated the detuning Δω as done in previously described phase-oscillator simulations. It has been shown that the frequency preference of gamma oscillations shifts as a function of input drive, both in experimental studies [13,14,78,79] and in computational studies of gamma-generating networks [14,23,41]. Therefore, one can manipulate input drive to change the frequency preference of a network and hence the detuning Δω. This relationship was exploited here to replicate the coherence and PLV findings from the phase-oscillator simulations. For manipulating detuning Δω, we altered the mean fixed input current to the E-cells of network Y (Fig 4C) while keeping input current fixed to network X. These manipulations are reported in Fig 4D and 4E. In addition, we will also show results from network simulations in which we changed the cross-network synaptic strengths. For manipulating coupling strength κ, we altered E→E_,_ connection strength or E→I connection strength (both excitatory AMPA type). These manipulations are reported in Fig 5B–5E.

#### Interacting networks receiving different levels of input-drive (detuning)

We first manipulated the detuning Δω by changing the input drive for a given fixed bi-directional coupling E-E and E-I coupling values (Fig 4A). In Figure B in S1 File we show the same results for the uni-directional connectivity case which gave similar results. The testing data were generated as described above (Fig 4B). To demonstrate that we could shift the oscillation frequency difference by changing the input drive difference, we plotted in Fig 4C the intrinsic frequency difference between network X and Y as a function of the input drive difference. Here, the networks were uncoupled to have an accurate measure of the intrinsic (natural) frequency difference. In Fig 4D, the coh^2^ and the PLV^2^ estimates are plotted as a function of excitatory input drive (ΔE-drive), where different lines represent different SNR levels (0.3, 2.2,4.8,9.1,36.2). The black line represents the PL (Fig 4D), which decreased rapidly with increasing excitatory input difference (ΔE-drive). The coh^2^ (left panel Fig 4D) showed strong overestimations particulary in the high SNR range and at higher ΔE-drive. By contrast, the PLV^2^ (right panel Fig 4D) converged with higher SNR towards the PL^2^ (computed from the noise free signals). With low SNR the PLV^2^ underestimated the PL^2^. In Fig 4E, we plot the information flow (TE) between network X and Y as a function of ΔE-drive. The information flow between the networks declined overall as a function of ΔE-drive in a manner very similar to the dependency of PLV^2^ on ΔE-drive, confirming that information flow can be highly dependent on oscillatory synchronization [18,21]. Note that the changes in information flow were achieved here without changing the synaptic coupling values, and relied purely on shifting the input frequency (E-drive) to the networks. Importantly, PLV^2^ tracked the synchrony-dependent information flow changes accurately, whereas coh^2^ appeared rather unrelated to information flow changes, particularly in the higher SNR range. We quantified these observations in Fig 4F by plotting the squared correlation coefficient (given an estimate of explained variance) between information flow TE measure and coh^2^ (black line) or between TE and PLV^2^ (red line), as a function SNR (in log scale). It can be seen that the explained variance in TE measured by coh^2^ is lower than by PLV^2^, particulary for the higher SNR ranges.

#### Interacting networks with different strengths of excitatory-to-excitatory and excitatory-to-inhibitory connections

We then explored the behavior of coh^2^, PLV^2^ and TE on testing data obtained after systematic and independent manipulation of the cross-network connections of E→I and E→E between network X and Y (Fig 5A). The detuning Δω was 2Hz (ΔE-drive = 2.5mV) for all conditions reported below. The power spectra were largely overlapping. Before describing the effects of the E→I and E→E connectivity manipulation, we will summarize the main findings. Remarkably, we found that E→I connections were powerful in tuning the phase synchronization strength between networks (increasing PrFM), whereas E→E connections only weakly changed phase synchronization strength, but induced robust PrAM in the network signals. This can be understood by considering that the I-cells neurons are thought to be critical in determining the gamma dynamics [39,80], hence E→I connections might be particularly well-placed for inducing synchronization between the networks. On the other hand E→E connections are less powerful for inducing synchronization, yet strongly modulate the spike probability of the receiving neurons. If the network oscillations are in-phase, E→E connections increase the spike-probability and hence the number of spikes in the receiving network, contributing to the gamma wave by giving it a higher amplitude. If the network oscillations are in anti-phase, E→E connections are not very effective in increasing spike-probability and instead they decrease the resulting gamma wave amplitude. Therefore PrAM was an inherent property of the oscillatory interactions compared to the phase-oscillator interactions. Note that despite the presence of PrAM, the phase synchronization dynamics could be well understood within the framework of the phase-oscillator model (TWCO framework) for the parameter ranges used here. We observed in these E→E and E→I manipulations that both types could have, in particular parameter ranges, a desynchronization effect. This is the case because E→E and E→I prefer synchronization at different preferred phase-relations. Hence, if both connections types are strong, there will be interference between the effects exerted by the two types of connectivity leading to an overall desynchronization. A detailed investigation of these phenomena goes beyond the scope of this paper. Of importance here is the question whether coh^2^ was able to represent robustly the PL^2^ and information flow TE in these different synchronization conditions.

To demonstrate the effect of connectivity manipulations between networks X and Y, we first show (in Fig 5B) an E→E connection strength manipulation (0mV– 0.02mV) for a fixed E→I connection strength (0.02mV). The top panel shows the information flow (TE), which decreased approximately monotonically with E→E strength. As shown in the middle panel of Fig 5B, the coh^2^ estimates increased monotonically with E→E strength. Similarly to TE, the PLV^2^ decreased monotonically with E→E strength. This striking example shows that in these configurations coh^2^ behaved opposite to PLV^2^ and information flow (TE).

In Fig 5C–5F, we show the behavior of TE, PLV^2^, coh^2^ as well as estimated PrAM strength as a function of both E→I and E→E connection strength (SNR of ~10). The TE increased for both E→I and E→E, but more strongly for the former (Fig 5C). When both were relatively strong the information flow was low. The same pattern could be observed with PLV^2^ (Fig 5D) indicating that phase synchronization to a large extent formed the basis for changes in information flow. By contrast, coh^2^ (Fig 5E) increased for both E→I and E→E, but more strongly in the latter case, hence showing a very different pattern compared to TE and PLV^2^. For all simulation conditions, we estimated PrAM (modulation strength in %, Fig 5F). PrAM clearly increased with increases in E→E strength, but not with increases in E→I strength. These results indicate that coh^2^ estimates substantially reflected changes in PrAM (which were less predicative for changes in information flow). We quantified these observations in Fig 5G and 5H. We computed the squared correlation coefficient between changes in PrAM and changes in coh^2^ (black line) as well as the between changes in PrAM and PLV^2^ (red line) as a function of different SNR (Fig 5G). The results show that coh^2^ strongly reflected changes in PrAM induced by E→E connections, with explained variance increasing with SNR. On the other hand PLV^2^ estimates did not explain variance in PrAM, with explained variance approaching 0 with increasing SNR. In Fig 5H we computed the squared correlation coefficient between changes in information flow (TE) and changes in coh^2^ (black line) as well as between changes in information flow (TE) and PLV^2^ (red line), again as a function of SNR. Here the picture was opposite. PLV^2^ reflected well the information flow, and even more so with increasing SNR. Coh^2^ on the other hand, could hardly explain any changes in information flow with values tending towards 0 when SNR was increased. This shows that spectral coherence in certain parameter regimes poorly reflects changes in oscillatory phase-locking as well as the associated changes in information flow.

### Discussion

In this study, we have demonstrated that spectral coherence exhibited serious problems over a large parameter range in quantifying phase-synchronization and its influence on information flow among oscillating neural networks. As an alternative approach for quantifying phase relations, we explored the behavior of a phase-locking value (PLV) method that is based on the reconstruction of the instantaneous phase. To derive phase information we used singular spectrum decompositions (SSD) followed by the Hilbert Transform. We will now discuss in more details the implications of the results.

### Can spectral coherence be used for quantifying neural phase synchronization?

Spectral coherence or magnitude squared coherence [26] has been and still is a very useful statistical measure for quantifying frequency-resolved linear interdependencies between neural signals. Its robustness against noise, its mathematically well-analyzed statistical properties as well as its availability in neural data analysis software (Fieldtrip [41], Chronux [42]) makes spectral coherence estimation attractive to many neuroscientists. However, the validity of coherence estimation relies on the principal assumptions of weak-sense stationarity and linear interdependency, which need to be fulfilled by the data. In neuroscience, spectral coherence is largely used to determine the interdependency or ‘phase consistency’ of neural oscillations in different frequency bands (e.g., delta, theta, alpha, beta and gamma) between cortical or subcortical regions. The intriguing hypothesis investigated by many neuroscientists is that the amount of phase consistency among synchronizing neural oscillators might have important consequences for information processing and transmission in the brain [17,18,52,80–82].

The fundamental reason why spectral coherence in a large number of conditions cannot robustly estimate the phase locking among synchronizing oscillations lies in the process of synchronization itself. According to the physical definition of synchronization, going back to Huygens’ first description of interacting pendulums nearly 350 years ago [7,44], synchronization is a process in which ‘oscillators mutually adapt their frequency’. In other words, oscillators synchronize towards a common frequency by influencing the instantaneous frequency (phase derivative) of each other. These mutual influences among oscillators are described by the phase response curve [45]. Hence, phase synchronization goes hand in hand with systematic frequency variations over time, which makes the process non-stationary. Therefore, it violates an essential assumption that is required for computing spectral coherence. The underlying problem of spectral coherence to estimate phase synchronization has its roots in the phase estimation of the signal, rather than in the quantification of the phase-relation distribution. In other words, the problem lies in what the phases represent that are derived from the Fourier cross-spectral density for a giving frequency. Therefore, the spectral coherence behavior reported in this paper can be generalized to any other spectral approach that uses a distribution of phases derived from the Fourier cross-spectral densities.

As we have shown in detail, the synchronization process leads to phase-relation dependent frequency modulations (PrFM) as well as potentially to phase-relation amplitude modulations (PrAM). These systematic modulations lead to modulation sideband peaks in the Fourier periodogram. These modulation sidebands lead to high coherence values, because a modulation sideband induced by oscillator X in the Fourier periodogram of oscillator Y shares frequency and consistent phase-relations with oscillation X itself. These modulation sidebands are well known from the cross-frequency coupling CFC literature [59,60], where interdependencies between frequency bands (e.g. between theta 3-7Hz [10,63,83,84] or alpha~ 8-12Hz [85,86] and gamma ~30-80Hz) are investigated. In particular, systematic power modulation of a higher frequency oscillation (e.g. gamma) as a function of the phase of a slower frequency oscillation (e.g. alpha) has been described [62,87]. This is similar to the PrAM effect described in this paper with the difference that PrAMs are induced by as a result of interactions between oscillations within the same frequency band. Further, the modulation is not a function of the phase of an oscillation, but a function of the phase-relation between two given oscillations.

In the present study, we show that the modulation sidebands induced by both PrFM and PrAM can strongly affect the spectral coherence estimate, because the Fourier estimated phases not only represent the phase of the oscillation, but also the phase of the modulation sidebands (PrFM, PrAM). Most relevant to experimental neuroscience, when oscillatory data were simulated using not only extrinsic (measurement) noise but also intrinsic (phase) noise, which lead to realistic, broad power spectra, the modulation sidebands still affected the coherence spectra. As a consequence, for each frequency of the Fourier cross-spectral density, the phase might represent the phase of the oscillation and/or the phase of the modulation sideband. In other words, the phase-relation distribution in this case did not reflect the true underlying phase-relation distribution between two given synchronizing oscillations, but a distribution modified by the presence of the modulation sidebands. We have demonstrated this phenomenon in abstract phase-oscillator models (which can be applied to any frequency band) as well as in detailed spiking neural network models (specific for gamma-band oscillations). In the latter neural network simulations, we showed that spectral coherence did not robustly reflect the synchronization-dependent information flow between two oscillating networks when input strength or connectivity strength was manipulated. Particularly disastrous for the idea that coherence is related to information transmission in the brain were the effects of changes in the strength of cross-network E→E synaptic connections, which modulated the magnitude of PrAM. This E→E connectivity manipulation firstly confirmed that spectral coherence is not a pure phase-locking estimate, but instead strongly reflects amplitude fluctuations. This held true even for the modified coherence formula that has been shown to be robust against amplitude correlation [41,53]. Secondly, we found that spectral coherence was weakly related to information flow. We would like to emphasize that the effects of PrAM should not be confused with the known effects of amplitude correlation on coherence [34,54], because PrAM still affects coherence if amplitude correlation is 0, as was the case in our simulations. This is because amplitude fluctuations in the form of PrAM only need to be present in one of two oscillators. The mixture of PrFM and PrAM with the true phase-locking between oscillators makes coherence values difficult to interpret, as shown by the weak relation between coherence and information transmission for certain conditions. Hence, it could be argued that spectral coherence often will not be the preferred method to test particular theories on phase relations and information transmission during neural oscillatory synchronization in brain networks.

### Generality of simulation and analysis results

The question can be asked whether the limitations revealed by our work for the use of spectral coherence for estimating phase locking is due to the use of the particular phase-oscillator model for generating simulated oscillatory data. However, the phase-oscillator model used here is a very general model (theory of weakly coupled oscillators, TWCO) and that the underlying synchronization theory [7] is widely accepted. It describes the core concepts of the phase response curve (PRC) and the Arnold tongue. These concepts are widely used for describing interactions of oscillations of various types including (noisy) limit-cycle as well as chaotic oscillations [7,50]. Moreover, synchronization theory is used in many scientific fields, for example, to describe the synchronization of electrochemical oscillations [88], of molecular circadian rhythms [89] among individual fireflies [90], and of climate oscillations [91]. In (theoretical) neuroscience these concepts are also well established [37,45,73,76] and have been used to understand synchronization properties of single neurons [45], the emergence of neural network oscillations [40] and their oscillatory interactions [52] and traveling wave properties [73]. Since neural networks should be seen as dynamical oscillating systems, similar in their basic characteristics to other dynamical systems in nature, we expect that the synchronization theory is a plausible and relevant framework for understanding neural oscillatory interactions. By the same token we expect that the results of the present study has general relevance for any type of neural oscillations in the brain, and perhaps also for the study of oscillations outside neuroscience. However, to further strengthen the methodological investigation of coherence (and the PLV approach), we also used a neural network model that generated gamma oscillations according to known neural principles [40]. This neural network represents a biophysical more plausible implementation of the principles shown with the phase-oscillator model. When using the simulated data from this neural network, we could confirm the results from the phase-oscillator model [40].

Another question that can be asked relates to potentials limitation of our work due to restricting our analysis to continuous network field signals. The effects of the various manipulations in our simulated data on Spike-Field coherence (SFC), which quantifies the locking of spike probability to a particular field oscillation phase, were not investigated. However, it can be expected that estimating the locking of spikes from spike trains in network X to the field signal from network Y will lead to similar problems for SFC as we have described for spectral coherence. This is because also here, the phase estimate from the field signal will be affected by modulation sidebands. These distorted phases will also be problematic for SFC. Further, phase-relation dependent amplitude fluctuations might also affect SFC estimates. Note that in neuroscience, other methods are used that assume weak-sense stationarity, such as frequency-resolved granger directionality analysis [92,93]. We expect that also these techniques might show problems due to the non-stationary nature of oscillatory synchronization.

### The partial synchronization state: Realistic for neuronal oscillatory data?

Using simulated data from both the phase oscillator model as well as neural network models demonstrated severe limitations of spectral coherence to accurately capture phase locking and information transmission across a broad range of conditions. Nevertheless, the question can be asked whether the non-stationary nature of the synchronization process that follows from it is biologically plausible and likely.

We have indeed stressed that the synchronization among neural networks (oscillators) is inherently non-stationary, and leads in a broad range of conditions to partial (intermittent) synchronization. In this regime, oscillators do have frequency differences (do phase precess), yet they still have preferred phase-relations that are reflected in non-uniform phase-relation distributions. In a complete or perfect phase-locking state, the phase-relation is constant (no phase precession) and the synchronized oscillators do not have a frequency mismatch. Why would the partially synchronized regime (being problematic for spectral coherence) be the most likely regime for neural oscillatory data? Even in the hypothetical case of noiseless empirical data, it would be unlikely (due to detuning) that synchronization would be perfect, and imperfect synchronization by definition leads to a complex state of partial synchronization, characterized by changes in phase relations and frequency among oscillators over time. In real empirical data, noise is inescapably present, and noise will further degrade the imperfect phase locking between oscillators (or neural networks approximated by oscillators) [7,52]. With noise we mean intrinsic (phase) noise that changes the instantaneous frequency of the oscillations. ‘Noise’ can for example be due to the inherent instability of the oscillation (e.g. low synchrony among neurons generating the network oscillation). Hence, given the noisiness and complexity of cortical networks we believe that the partially synchrony regime can be expected to be the dominant regime. These theoretical considerations are supported by experimental data. A few studies have directly investigated and shown partial (intermittent) synchronization in cortical activity [32,94]. Moreover, there is indirect evidence from studies showing that for cortical gamma oscillations the frequency and amplitude evolution over time is noisy and complex [30], and changes as a function of cortical state [71,85]. Neuronal oscillations in general behave rather like very noisy limit-cycle oscillations or chaotic oscillations, and clearly not like noiseless oscillators [95]. Furthermore, several experimental papers have indicated phase-locking among neural oscillations of slightly different frequencies [8,12,13] indicating that detuning among interacting neural oscillation does exist. Another study on cortical gamma oscillation showed synchronization and phase precession at the same time [17] indicating a state of partial synchronization. Therefore, theoretical as well experimental indications suggest that partial synchrony should be expected as the most likely regime.

### Testing the underlying assumptions of spectral coherence

The concept of systematic frequency variation and frequency differences among oscillations within a ‘frequency band’ has only recently attracted scientific interest [23,31,78,79,96]. This might explain why the problem of frequency variation of neural oscillatory signals for stationary methods like spectral coherence is still not commonly recognized. It is striking that till today most research studies using spectral coherence are published without including a test of the assumption of weak-sense stationarity (nor linearity), despite that experimental and theoretical work suggests that neuronal oscillations are likely to be non-stationary. Whether one assumes a phase synchronization model or not underlying oscillatory interactions, it is important to validly test for weak-sense stationarity (or at last have good reasons to assume stationarity) before applying spectral coherence on neural signals. A reason this is rarely done might be that appropriate tests of weak-sense stationarity are not well-known in the neuroscientist community and not always available in popular software packages. The weak-sense stationarity assumption states that the auto-correlation function of a process should not systematically change over time. If for example an oscillatory signals has periods of higher frequency followed by periods of lower frequency, the signal violates the weak-sense stationarity assumption. Methods have been proposed [27,97] that statistically test the null hypothesis of weak-sense stationarity by quantifying the variability in time-frequency representation of a signal and by testing whether it deviates from expected variation of a stationary random process.

The testing of stationarity in empirical data is necessary assuming that the analysis is directed at single trial data. It is only in single trial data that the detailed non-stationary interactions can be appropriately quantified. Yet, it often is common practice to justify the use of spectral coherence based on the stability of the trial-averaged TFR. For example, a stimulus onset trial-averaged TFR [98] shows often transients shortly after stimulus onset and then it looks rather stable. Therefore, the transients are typically excluded from analysis, after which spectral coherence is applied on the ‘sustained part’ of the (trial-averaged) TFR. Importantly, trial-averaging eliminates all frequency or power variations that are not strictly locked to the stimulus-onset (or the event to which the data are aligned). An example of variation lost by trial averaging is the presence of effects induced by saccadic or microsaccadic eye movements during presentation of a stimulus. It is known that saccadic eye-movements strongly affect the oscillatory properties [29,71,99,100] in the ‘sustained part’ of the trial-averaged TFR of signals in visual cortex, and that these saccadic effects can be useful indicators of perceptual and cognitive states [101]. However, these TFR variations are not locked to stimulus onset, but to saccades which occur at various time points within a trial, and therefore these interesting single-trial TFR variations are removed by classical stimulus-onset triggered trial averaging. Hence, trial-averaged TFR cannot be used to assess whether signals in certain periods are stationary or not.

Another condition that needs to be satisfied for spectral coherence methods to be applied in a valid manner is linearity. In the present study, the phase coupling in the phase-oscillator model as well as in the neural network model was linear. This was evident in phase-phase plots of pairs of oscillators, which exhibited clear straight lines indicating that the phases were indeed linearly related. Hence, in our simulated data the linearity assumption for the application of spectral methods was satisfied. However, similarly to the weak-sense stationarity assumption, the nature of phase coupling should be investigated in empirical data before using linear methods such as spectral coherence or the phase-locking value. Moreover, methods derived from information theory are suitable for linear as well non-linear interactions [102] and should be used by default if one does not know whether phase relationships are linear.

### Alternative phase locking estimation approaches

The problem of non-stationarity in neuronal signals has been previously reported [33–35,103,104] and several alternative methods, based on instantaneous phase, have been proposed and successfully used. Below, we will give a short overview of possible strategies to estimate instantaneous phase in experimental multi-component signals.

It is noteworthy that the concepts of ‘instantaneous phase’ and particularly ‘instantaneous frequency’ could be regarded with skepticism, because of the time-frequency uncertainty principle [105], and because instantaneous phase or frequency cannot be easily defined for signals like those observed in the brain. It is correct that in a TFR, higher time resolution leads to lower frequency resolution, and vice versa. At the same time, the higher the time resolution, the more the estimated phase will approximate the instantaneous phase, at the cost of low frequency resolution. Hence it is impossible to get an accurate estimate of both phase and frequency at the same time. However, the instantaneous frequency as a mathematical concept is nothing else than the derivative of the instantaneous phase, and hence, if instantaneous phase is known then the instantaneous frequency can be. Nevertheless, instantaneous phase or frequency can only meaningfully be defined if the signal is mono-componential (e.g., not mixed with two or more oscillations, trends or transients). That means that the signal must be well described by a single peak frequency at each point in time. Because neuronal signals (like the local field potential) usually consist of many components, a decomposition technique of some sort must precede the estimation of instantaneous phase, such that phase estimates can be linked with a well-defined frequency component. Further, instantaneous phase estimations have their own challenges. For example, they can be affected by amplitude variation properties of the signal [106] and are often more sensitive to noise leading to phase slips [55]. In practice, TFR techniques approximating instantaneous phase might give appropriate estimates for most cases [35].

There are two basic analysis strategies from which the instantaneous phase can be approximated, one based on complex TFR representations (based on wavelet or short-time Fourier transform) and the other based on the Hilbert Transform (HT) preceded by a decomposition of the signal. The first strategy convolves the signal with e.g. complex wavelets [35] to estimate the complex wavelet TFR. From each complex value in the time-frequency plane the phase can be estimated. The advantage of the approach is that it is relatively robust against noise and that wavelets are well understood. Disadvantages are the lower time resolution of the phase trace and that the selection of points in the TFR for reconstructing the phase trace is not straightforward. Furthermore, the decomposition is linear [36]. The second strategy uses the Hilbert Transform (HT) [105], which converts a real-valued signal into a complex analytical signal from which the instantaneous phase can be extracted. The instantaneous phase is only well defined if the signal is mono-componential [57,107,108]. Oscillatory brain data are multi-componential and therefore these signals need to be decomposed. One approach is filtering the signal in a predefined frequency range in which most of the oscillatory power is expected to be. This approach is often used in neuroscience, for example for estimating cross-frequency coupling CFC [60,62,85,102]. An advantage of filtering is computational efficiency, yet with the disadvantage of fixed frequency borders, defined by the researcher based on pre-knowledge. Another approach is based on empirical mode decomposition EMD [57] which decomposes in a data-driven manner the signal into intrinsic mode functions (IMF), which are well-suited for applying the HT. Similarly, singular spectrum decomposition (SSD) decomposes the signal in oscillatory narrow-band components on which HT can be applied [36,56]. An advantage of SSD is that it has good de-mixing properties [36], assuring that signals are narrow-banded. Moreover, the SSD algorithm has been optimized to be useable for neurophysiological datasets [56]. In this study, we used the SSD approach in combination with HT to reconstruct the instantaneous phases.

Our results showed that PLV estimates based on the instantaneous phase reconstructed by SSD-HT gave more robust estimates of the true underlying phase-locking values and reflected better variations in synchronization–dependent information flow between neural networks compared to spectral coherence. The estimates were robust against amplitude fluctuation in the form of PrAM, although small effects of PrAM could be observed in the low SNR regimes. Although PLV could severely underestimate phase locking for lower SNR, increasing SNR allowed the PLV to approach the expected phase locking value. Moreover, in terms of explained variance of information flow changes between networks, the PLV estimates were superior to coherence for any SNR investigated. We expect similarly superior results for all approaches that are based on reconstruction of the instantaneous phase (based on the use of wavelets or the Hilbert transform preceded by some decomposition technique). Finally, although there are conditions in which spectral coherence might be appropriate, methods that combine appropriate signal decomposition with instantaneous phase reconstruction permit a more detailed and accurate look on time-dependent changes in synchronization properties of neural signals. This provides definite advantages when trying to determine neural network mechanisms underlying perception, cognition, and behavior.

## Supporting Information

S1 File Supplementary Information and Simulation Codes. The supplementary file contains supplementary methods, including mathematical derivation of phase-locking strength between two-coupled phase-oscillators, a more detailed description of the modulation sidebands in the FFT induced by systematic frequency or amplitude fluctuations and more information about the singular spectrum decomposition (SSD) (Methods A). The file contains two figures. A schematic description of the different phase-locking approaches used in this study (Figure A). Analysis of unidirectionally interacting gamma-generating neural networks receiving different detuning levels (Figure B). The file contains also two Matlab simulation codes. Phase-oscillator simulation (Simulation Code A) and simulation of gamma–generating neural network (Simulation Code B).
(DOCX)
