# Supplementary material for: Quantifying Neural Oscillatory Synchronization: A Comparison between Spectral Coherence and Phase-Locking Value Approaches
Source: PLoS One. 2016 Jan 8;11(1):e0146443. doi: 10.1371/journal.pone.0146443 (PMC4706353; doi:10.1371/journal.pone.0146443)
Supplement: S1 File — The supplementary file contains supplementary methods, including mathematical derivation of phase-locking strength between two-coupled phase-oscillators, a more detailed description of the modulation sidebands in the FFT induced by systematic frequency or amplitude fluctuations and more information about the singular spectrum decomposition (SSD) (Methods A). The file contains two figures. A schematic description of the different phase-locking approaches used in this study (Figure A). Analysis of unidirectionally interacting gamma-generating neural networks receiving different detuning levels (Figure B). The file contains also two Matlab simulation codes. Phase-oscillator simulation (Simulation Code A) and simulation of gamma–generating neural network (Simulation Code B). (DOCX) [file pone.0146443.s001.docx]

**Supporting File S1**

**Methods A**

**Deriving phase-locking from two coupled phase-oscillators**

In this study we used a commonly applied measure to test for a non-uniform phase-relation distribution which is defined as the mean resultant vector length.

$$\left(1\right) \text{PL}=\left| \frac{1}{T }\sum_{t=1}^{T} \text{e}^{\text{iθ}\left(\text{t}\right)}\right|$$

where *T* is number of sample points and *t* is the time variable. If the phase-relation distribution is uniform (each phase has equal probability), the PL will give a value of 0 [7], while if there is a particular peak in the distribution, the PLV will give a value > 0. There are also different measures that might be more favorable [100].

The true phase locking value PL between two coupled oscillators is related in a simple manner to the sinusoidal coupling function defining the PRC [70]. If the detuning |∆ω| is smaller or equal to the amplitude of the coupling function $\kappa$ then the phase locking value is 1. This is the case when full synchrony among oscillators is achieved. In this case, the instantaneous phase relation is constant over time and no phase precession is present. This is because the detuning ∆ω and the associated tendency to phase precess, cannot overcome the ‘attractor’ defined by the PRC with the strength κ. The true phase locking value PLV_T_ then equals to:

$$\left(2\right)\text{ PL=1 } \text{if} \left|\Delta\text{ω}\right|\leq\kappa\forall t>t_{0}$$

However, in the case in which frequency detuning ∆ω(t) is stronger than the coupling strength ($\kappa$: ∆ω(t) >$\kappa$ ), the attractor is not strong enough to prevent phase precession. Hence, full synchrony is no longer possible. Yet, the phase relation distribution is not uniform and oscillators are still more likely to be in their preferred relationship. This is because the rate of phase precession (or instantaneous frequency difference) changes as a function of phase-relation. When the oscillators are at their preferred relationship, the phase precession speed is minimized (the point of the PRC with the strongest phase adjustment opposite to the phase precession), whereas at the non-preferred phase-relation, the phase precession speed is maximal. The modulation of the instantaneous phase relation leads to non-uniform phase-relations and thus to phase-locking of >0.

For deriving the PL for |∆ω| >κ, we computed the resultant vector length of the phase-relation probability density distribution (pdf), which can be derived from the Adler equation (see equation 3) that describes the time derivative of the phase-relation as a function of the phase-relation and a frequency difference constant. Notice that the time derivative of the phase-relation is the instantaneous frequency difference. In the case of |∆ω| > κ the time derivative is always non-zero (no fixed point). To derive the pdf, we first rearranged the terms of the Adler equation (see main manuscript equation 6) to

$$\left(3\right)\text{ } dt=\frac{1}{\Delta\omega+\kappa\text{sin}\left(\theta\right)} d\theta$$

where the time derivative is a function of phase derivative and the inverse of the Adler function. Integrating this function over the phase space gives the characteristic time period T for which the oscillators need to make a full phase precession [7]. Because the (noiseless) oscillators repeat exactly the same patterns at intervals T, considering only period T is sufficient for the full determination of the pdf.

$$\left(4\right)\text{ }T=\int_{0}^{2\pi} \frac{1}{\Delta\omega+\kappa\text{sin}\left(\theta\right)}d\theta$$

It can be easily seen that integrating around a small phase interval, divided by T, gives the probability value (the amount of time the phase-relation θ spent in the phase interval [a,b] within the time scale T).

$$\left(5\right)\text{ }\Pr\left[a \leq\theta\leq b\right]= \frac{1}{T}\int_{a}^{b} \frac{1}{\Delta\omega+\kappa\text{sin}\left(\theta\right)}d\theta$$

The inverse of the Adler function therefore describes the probability density function.

$$\left(6\right)\text{ }pdf\left(\theta\right)=\frac{1}{T}\frac{1}{\Delta\omega+\kappa\text{sin}\left(\theta\right)}$$

To compute the true phase-locking value PLV_T_, one needs to integrate over all the phases with their associated probability densities.

$$\left(7\right) \text{PL}=| \int_{0}^{2\pi} pdf\left(\theta\right) e^{i\theta} d\theta| \text{if} 0\langle\kappa\langle|\Delta\text{ω}|$$

Complete asynchrony is only possible when the oscillators are not coupled and hence there are no phase-relation dependent modulations of phase precession speed (pdf is described by a constant).

**Modulation sideband induced by PrFM**

For demonstration purposes, we assume as a first step that the oscillatory processes have no intrinsic (dynamical) and extrinsic (measurement) noise. Further, we assume that oscillator $X$ is unperturbed and unidirectionally coupled to oscillator Y with a coupling strength of κ=1. Oscillator Y exhibited PrFM. The oscillators have a frequency difference such that | $\omega_{X }$- $\omega_{Y}$| > κ. The parameter $\varepsilon$ is the initial phase-offset between the oscillators. The discrete phase-evolution of oscillator X and oscillator *Y* is defined as follows:

We now demonstrate that the (true) PLV between the oscillators is equal to the absolute Fourier transform (amplitude) of the modulated oscillator Y evaluated at frequency ω_X_. The PLV equation can be changed to the equivalent form of:

The absolute (discrete) Fourier coefficient (amplitude), evaluated at discrete frequency ω_X_, can be formulated as follows:

with = 2π$\omega_{X}$t

where we transformed the discrete phase evolution ϕ_Y_(t) of oscillator Y into an equivalent complex notation (complex sinusoid with unit amplitude) Y(t). It can easily be seen that equation 11 and 12 are equivalent.

$$\left(13\right)\to|S_{Y}\left(\omega_{X}\right)|= \text{PL}$$

Coherence between S_X_(ω_Y_) and S_Y_(ω_X_) will be 1 assuming that the modulation function does not vary across trials (consistent preferred relationship across trials/time), because the relationship between S_X_(ω_Y_) and S_Y_(ω_X_) is then consistent independent of the actual PL. Equation 13 shows that the phase-locking strength between oscillator X and the modulated oscillator Y is equal to the amplitude induced in the magnitude spectrum of oscillator Y at the frequency of ω_X_, hence the amplitude of the modulation sideband. In other terms, if amplitude of the oscillation is unity (=1) and no noise is present, then the amplitude of magnitude spectrum at frequency ω_X_ is equal the phase-locking, e.g. PLV of 0.4 will give a magnitude of 0.4 in the magnitude spectrum. Generally, we derived the modulation sideband amplitude (in the case PrAM=0) as follows:

$$\left(14\right) \text{SM}_{\text{PrFM}}=A \text{PL}$$

where *A* is the amplitude of the oscillator that is modulated.

**Modulation sideband induced by PrAM**

The relation between PrAM and modulation sideband can be shown using trigonometry, assuming no phase-locking, as follows [56]:

$\left(15\right) X\left(t\right)=\sin\left(\omega_{X}t+ \varepsilon\right)$

$$\left(16\right) Y\left(t\right)=\left(1+\cos\left(\Delta\text{ωt}+\varepsilon\text{ }\right)\right) \text{sin}\left(\omega_{Y}t\right)$$

$$\text{with }\Delta\text{ωt}=\omega_{Y}t-\omega_{X}t$$

The cosines function represents the phase-relation dependent amplitude modulation function (PrAM). The variable ε is the phase-relation offset between the two oscillators. The equation Y(t) can be rewritten in the following form:

$$\left(17\right) Y\left(t\right)=\sin\left(\omega_{Y}t\right)+\frac{1}{2}\sin\left(\omega_{X}t+\varepsilon\right)+\frac{1}{2}\sin\left(\omega_{Y}t+\Delta\text{ωt}+\varepsilon\right)$$

$$\text{with} \omega_{X}\text{t}=\omega_{Y}\text{t}-\Delta\text{ωt}$$

The second and third term represent the two symmetric (±∆ω) modulation sidebands. Because the second term, the modulation sideband at frequency ω_X_ of oscillator Y, representing the amplitude modulation function, the coherence at frequency ω_X_ between oscillator X and Y will be 1, despite a true PLV of 0, because the phase-relation offset ε does not change the phase relation between the modulation sideband of oscillator Y and oscillator X. The phase-relation offset ε affects them both in the same way.

If no PLV is present (no PrFM), the amplitude of the modulation sideband can be computed as:

$$\left(18\right) \text{SM}_{\text{PrAM}}=A\frac{\alpha}{2}, \text{if} \text{PL}=0$$

where A is the mean amplitude of the oscillator and α the modulation strength of the PrAM. Because PL is 0 (phase-relation distribution is uniform) each phase-relation is equally likely and the weighting (in terms of amplitude) is alone defined by the PrAM cosine modulation function.

**Modulation sideband induced by PrAM and PrFM combined**

If both PrAM and PrFM are present, we derive the amplitude of the modulation sideband as follows:

$$\left(19\right) \text{SM}_{\text{PrFM, PrAM}}=A\left|\int_{\theta=-\pi}^{\theta=\pi} \alpha\text{cos}\left(\theta\right) \text{pdf}\left(\theta\right)e^{i\theta}d\theta\right|$$

where pdf(θ) is defined as in equation (6), α is the modulation strength of PrAM, θ is the phase-relation between the two oscillators, *A* represents the mean amplitude of the modulated oscillator. The amplitude of the modulation sideband SM can be understood as the resultant vector length where each phase-relation defines a vector of angle θ and the vector length defined by its probability (defined by pdf(θ)) and its relative amplitude (defined by αcos(θ)) scaled by the overall mean oscillator amplitude A.

In the case of only PrAM, the vector lengths are alone defined by the amplitudes modulated by PrAM. In the case of PrFM only, the vector lengths are alone defined by the phase probability which is non-uniform as PL >0. If both PrAM and PrFM are present, the vector lengths are defined by both phase probability and amplitude.

**Singular spectrum decomposition (SSD)**

Given a zero-mean time series *x(n), n = 1,…,N*, the SSD algorithm iteratively extracts a set of component series from *x(n)* until the total variance of the extracted components reaches a user-defined threshold.

Each iteration consists of the following steps:

**Embedding**. The time series *x(n)* is embedded in a vector space of dimension *M*. Given an embedding dimension *M*, with *1 < M < N*, the embedding procedure forms *M* lagged vectors *xi = (x(i),…,x(N)*; *x(1),…,x(i-1))*, with *i = 1,…,N - M + 1*. For instance, given the time series *x(n)* = {1, 2, 3, 4, 5}, and an embedding dimension *M* = 3, the corresponding trajectory matrix will be:

$$\left(20\right) X=\left[\begin{matrix} 1 & 2 & 3 & 4 & 5 \\ 2 & 3 & 4 & 5 & 1 \\ 3 & 4 & 5 & 1 & 2 \end{matrix}\right]$$

such that this is a Hankel matrix (constant cross-diagonals) of size (*M x N)*. This embedding procedure is different from the standard one used by SSA, and it has the advantage of enhancing its oscillatory content. Possible discontinuities generated by the periodic extension in matrix *X* are negated by projection on the subspace of the principal components. The embedding dimension *M* is automatically estimated as *Fs/f*max, with *f*max being the dominant frequency in the power spectral density (PSD) of *x(n)*, and *Fs* the sampling frequency.

**Decomposition**. The singular value decomposition of the trajectory matrix *X* is then computed, providing *X = UDV^T^*, with *U = (M x M)* and *V = (N x N)* being orthogonal matrices containing the left and right singular vectors, respectively, and *D = (M x N)* being a matrix containing the singular values on the main diagonal and zeros elsewhere.

**Grouping**. Out of the *M* principal components of *X*, a subset *(P<M)* is selected that corresponds to a dominant frequency in the range *[f*max *- δf; f*max *+ δf]*. Practically this is determined by selecting the components whose left eigenvectors show a dominant frequency in that range. This allows the component series, which will be reconstructed from this subset, to describe a well-defined time scale. The width of the dominant peak *δf* is estimated by means of a Gaussian interpolation of the power spectral density of the time series *x(n)*.

**Reconstruction**. Only the selected *P* principal components are used to generate a rank-*P* approximation of *X*. The corresponding time series is then reconstructed by suitable diagonal averaging of this matrix along the cross-diagonals. In order to carry out the average along the *i*-th cross-diagonal of *X*, the wrapped part of the right hand block must be correctly appended to the top right of the left hand block. For the example above:

$$\left(21\right) \text{X}= \left[\left.\begin{matrix} & & 1 \\ & 1 & 2 \\ 1 & 2 & 3 \\ 2 & 3 & 4 \\ 3 & 4 & 5 \end{matrix}\right|\begin{matrix} & \\ & \\ 4 & 5 \\ 5 & * \\ * & * \end{matrix}\right]$$

where the asterisks mark the former locations of the elements composing the wrapped part moved to the right top of the left hand block. With this approach, each cross-diagonal contains the same number of elements *M* (with *M* = 3 in this example; the empty locations should be ignored).

The estimated component series is then subtracted from *x(n)*, and the procedure is iterated for the residual until a stopping criterion is met, when the energy of the residual falls below a pre-defined threshold.

**Supplementary Figures**

**
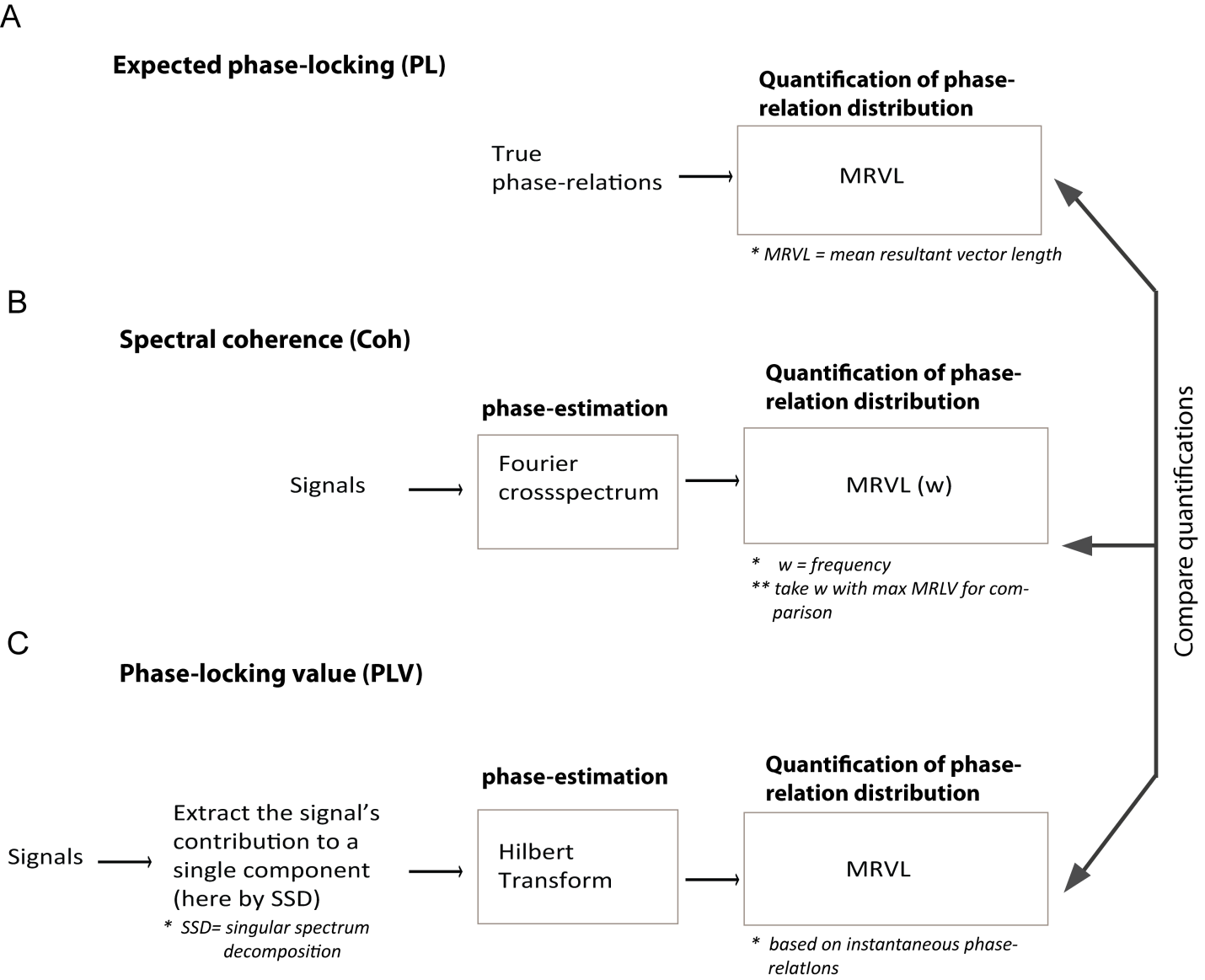
**

**Figure A**. **An overview of phase-locking estimation methods and interrelations.**

(A) The expected phase-locking and the comparison value for the estimation methods was the mean resultant vector length (MRVL) of the true phase-relations. (B) Schematic description of spectral coherence. Time-domain signals are transformed into the Fourier-domain. The phase-relation of a given frequency is derived from the Fourier cross-spectrum. (C) Schematic description of the phase-locking value (PLV). First it was ensured that the time-domain signals in the frequency range of interest (e.g. gamma-band) were mono-componental, here by applying singular spectrum decomposition (SSD). Then the Hilbert transform was applied to derive the instantaneous phase. As in( B) we used then the MRVL as a quantification of the phase-relation distribution.


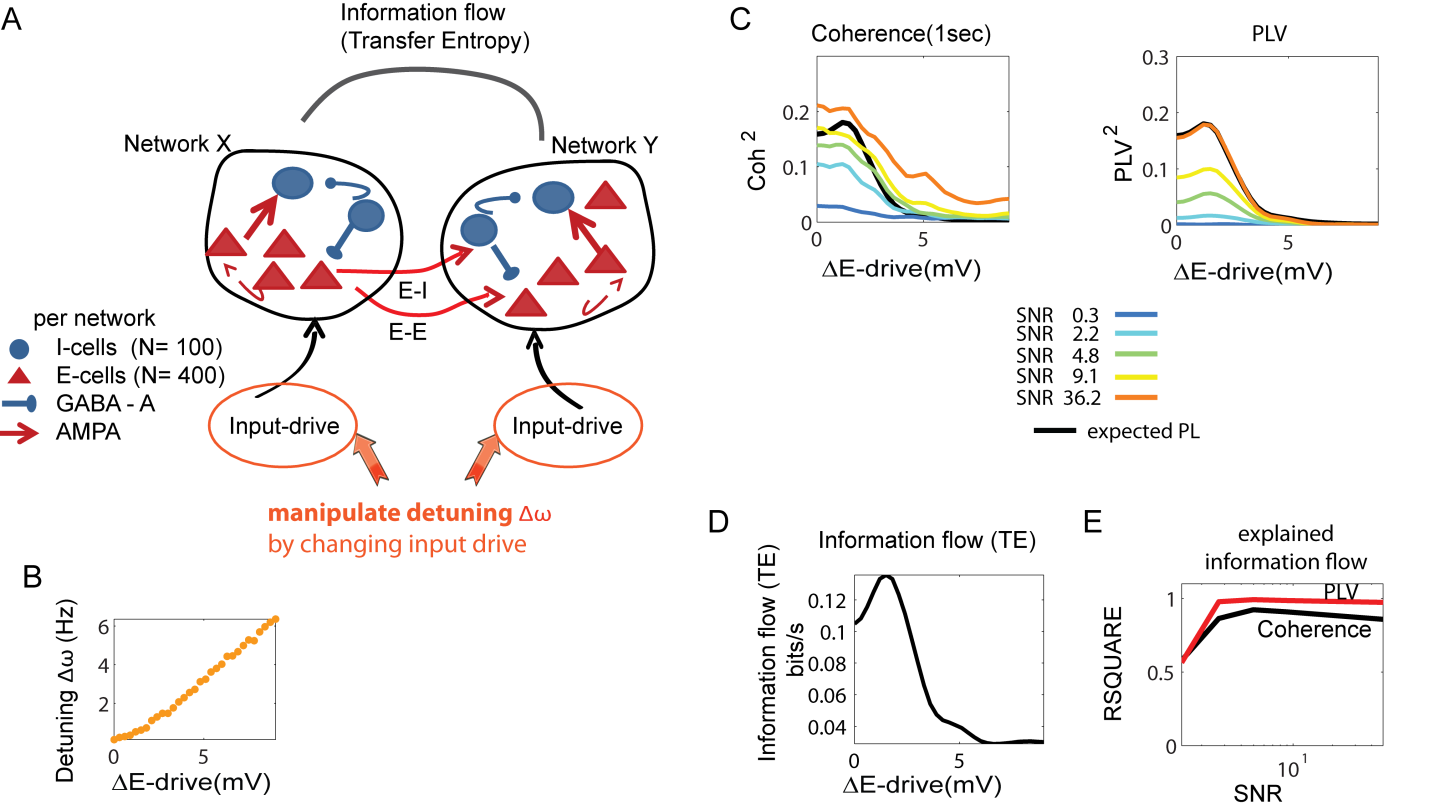


**Figure B.** **Network architecture similar to Figure 4, but with unidirectional connectivity from network X to Y.**

(A) Network architecture. (B) The detuning (intrinsic frequency difference) as a function of excitatory input drive difference. (C) The estimation of the phase-locking using spectral coherence (left) and PLV (right) as a function of excitatory drive difference for different SNR. The expected phase-locking PL was approximated by the PLV computed on traces without added measurement noise. (D) The transfer entropy as a function of excitatory difference. (E) The correlation squared coefficient between transfer entropy and coherence (black line) or PLV (red line) as a function of SNR.

**Simulation Code A: Phase-oscillator simulation code (MATLAB)**

function [parameter,data ,true_inst_ph]=phase_oscil_plv_plos(cfg)

%%%%%%%%%%%%%%%%%%%%%%%%%%%%%%%%%%

%Generates the data (two coupled phase osicllators)

%and estimates the FFT-PLV

%Output is generated to be easy to use for fieldtrip functions

% cfg.SNR white noise level added to the signal (measurment noise).

% If intrinisc noise is added, then this SNR definition will differ from the

% relative power ratio, because power is spreaded over frequencies.

% cfg.trial_number number of trials

% cfg.coupling symmetric coupling values between phase oscillators (coupling function is sinusoidal (defining phase response curve)

% cfg.detuning intrinsic (natural) frequency difference between oscillators

% cfg.triallength length of the simulation trial

% cfg.centerfrequency main frequency of the oscillations

% cfg.scale_intrinsic_noise scale factor of added (intrinsic) pink noise

% Example code =

% cfg=[];

% cfg.SNR=250;

% cfg.trial_number=100;

% cfg.coupling=0.5;

% cfg.detuning=3;

% cfg.centerfreq=35;

% cfg.triallength=1;

% cfg.scale_intrinsic_noise=0.05;

% [parameter,data , true_inst_ph]=phase_oscil_plv_plos(cfg)

% figure('Color','w'),plot(data.trial{1}(1,:),'k')

% xlabel('Time (ms) ')

% parameter

%%%%%%%%%%%%%%%%%%%%%%%%%%%%%%%%%%%%%%%%

if isfield(cfg,'SNR')

snr2 =cfg.SNR;

else

snr2 =1000;

end

if isfield(cfg,'trial_number')

trial_num =cfg.trial_number;

else

trial_num=100;

end

if isfield(cfg,'coupling')

coupling2 =cfg.coupling;

else

coupling2 =0.75;

end

if isfield(cfg,'detuning')

detuning=cfg.detuning;

else

detuning=3;

end

if isfield(cfg,'centerfreq')

centerfreq=cfg.centerfreq;

else

centerfreq=40;

end

if isfield(cfg,'triallength')

triallength=cfg.triallength;

else

triallength=1;

end

if isfield(cfg,'scale_intrinsic_noise')

scale_noise=cfg.scale_intrinsic_noise;

else

scale_noise =0;

end

%%%%%%%%%%%%%%%%%%%%%%%%%%%%%%%%%%%%%%%%%%%%

snrs=0;

mfr= centerfreq; %main frequency of the first oscillator

for snr=[ snr2]

snrs=snrs+1;

freqlin=[ mfr-detuning] % frequency of the second oscillator

nn=0;

for ffr=freqlin

nn=nn+1;

trt=0;

for trial=1:trial_num

number_of_nodes=2; % Two oscillators

initial_phase= [ randn(11)*2 randn(11)*2]; % Randomize initial phases

tim_sec=triallength;

time_steps=tim_sec+2; % The two extra seconds is due to transient dynamics at the beginning

dt=0.001; % step size (here 1ms)

phases = zeros(number_of_nodes,time_steps./dt);

phases(1:2,1)= initial_phase(1:number_of_nodes,1).*1;

clear noiseterm

for ind=1:2

noiseterm(ind,:)=powernoise(1,(time_steps./dt)+1,'normalize').*scale_noise;

end

f2=0;

for freqs2= ffr

f2=f2+1;

cops=0;

for coupling =coupling2

cops=cops+1;

K= [ coupling coupling];%coupling matrix (here symmetric)

W= [ mfr freqs2];

W=W.*(2*pi); %radians per sec

K=K.*(2*pi); %scaling of coupling

%%%%%%%%%%%%%%%%%%%%%%%%%%%%%%%%%%%%%%%%%%%%%%%%%%%%%%%%%%%%%%%%%%%%%%%%

for time=2:time_steps./dt %%% SIMULATION of the phase-oscillators %%%%%%%%%%

for ind=1:number_of_nodes

phases(ind,time)= phases(ind,time-1) + (dt*(W(ind))+ sum(dt.*K(:,ind).* -(sin((phases(ind,time-1) -phases(:,time-1)))) ) )+ noiseterm(ind,time-1) ;

end

end

%%%%%%%%%%%%%%%%%%%%%%%%%%%%%%%%%%%%%%%%%%%%%%%%%%%%%%%%%%%%%%%%%%%%%%%%%%%%%

ph= (mod(phases',2*pi))';

xx=(exp(1i*(ph(:,1:1:end)))');

%%%%%%%%%%%%%%%%%%%%%%%%%%%%%%%%%%%%%%%%%%%%%%%%%%%%%%%%%%%%%%%%%%%%%%

%%% NOISE %%%%%%%

noiseterm=randn(2,time_steps*1000).*((tim_sec*1000)/(2*sqrt(tim_sec*1000))); % white noise properly scaled

%%%%%%%%%%%%%%%%%%%%%%%%%%%%%%%%%%%%%%%%%%%%%%%%%%%%%%%%%%%%%%%%%%%%

trials{trial} = ((real(xx)'))+((noiseterm).* 1./sqrt(snr)); %creating composite signal

true_inst_ph{trial} =ph(:,2001:end);%

times{trial}=0.001:0.001:time_steps*1;

trials{trial} = trials{trial}(:,2001:end); % ecluding the first seconds

times{trial}=times{trial}(:,2001:end);

xx2=(exp(1i*( circ_dist(ph(1,2001:1:end),ph(2,2001:1:end)) ))'); %computation of phase dif on noisefree signal

trialx(:,trial)=((xx2));

disp(num2str(trial))

end

end

end

end

data=[];

data.trial=trials;

data.time=times;

data.label={'A','B'};

%%%%%%%%%%%%%%%%%%%%%%%%%%%%%%%%%%%%%%%

freq1=fft_perform(data); % performing FFT

%%%%%%%%%%%%%%%%%%%%%%%%%%%%%%%%%

allfrs(:,:,nn,snrs)= squeeze(mean(((freq1.fourierspctrm(:,:,:)).*conj((freq1.fourierspctrm(:,:,:)))))) ; %computing power

%%%%%%%%%%%%%%%%%%%%%%%%%%%%%%%%%%%%%%%%%%%%%%%%%%%%

PLV_estimate=PLV_perform(freq1); % computing phase locking value

%%%%%%%%%%%%%%%%%%%%%%%%%%%%%%%%%%%%%%%%%%%%%%%%%%%%

allcoh3(:,nn,snrs)= PLV_estimate; % FFT-PLV

%% the trials are concatenated before estimating PLV

allcoh2(nn,snrs)=abs(mean(trialx(:))); %% Approx. 'True value', here numerically estimated (but very close to analytically derived ones)

SNR(nn,snrs)=snr;%(1/snr).^2;

end

cohest=max(allcoh3); % find the maximum peak in the PLV spectra

cohest=squeeze(cohest);

if 0 %plotting

figure('Color','w','Position',[300 300 250 200])

subplot(1,1,1,'Fontsize',17)

plot(SNR,cohest,'Linewidth',2.5,'Color', [ 0 0 0 ])

axis tight

figure('Color','w','Position',[300 300 250 200])

subplot(1,1,1,'Fontsize',17)

plot(10.*log10(SNR),cohest,'Linewidth',2.5,'Color', [ 0 0 0 ])

axis tight

end

parameter.expected_locking =allcoh2;

parameter.fftestimate=cohest;

parameter.SNR=SNR;

parameter.trials=trial_num;

parameter.intrinsic_noise=scale_noise;

function [ft] = fft_perform(lfp)

Fs=1000;

trial_number= length(lfp.trial);

for tr=1: trial_number

L=length(lfp.trial{tr});

y=(lfp.trial{tr});

wins=hanning(length(y));

Y = 2.*(fft(y,L,2)./L);

f = Fs/2*linspace(0,1,L/2+1);

ft.fourierspctrm(tr,:,:) = Y(:,1:L/2+1);

end

ft.freq=f;

function r = circ_dist(x,y)

%

% r = circ_dist(alpha, beta)

% Pairwise difference x_i-y_i around the circle computed efficiently.

% Input:

% alpha sample of linear random variable

% beta sample of linear random variable or one single angle

% Output:

% r matrix with differences

% References:

% Biostatistical Analysis, J. H. Zar, p. 651

% PHB 3/19/2009

% Circular Statistics Toolbox for Matlab

% By Philipp Berens, 2009

% berens@tuebingen.mpg.de - www.kyb.mpg.de/~berens/circStat.html

if size(x,1)~=size(y,1) && size(x,2)~=size(y,2) && length(y)~=1

error('Input dimensions do not match.')

end

r = angle(exp(1i*x)./exp(1i*y));

function [PLV] = PLV_perform(ft)

crossspectra=squeeze(ft.fourierspctrm(:,1,:)).*conj(squeeze(ft.fourierspctrm(:,2,:))) ;

autospectra1=squeeze(ft.fourierspctrm(:,1,:)).*conj(squeeze(ft.fourierspctrm(:,1,:))) ;

autospectra2=squeeze(ft.fourierspctrm(:,2,:)).*conj(squeeze(ft.fourierspctrm(:,2,:))) ;

PLV=abs(mean(crossspectra./sqrt(autospectra1.*autospectra2),1));

function x = powernoise(alpha, N, varargin)

% Generate samples of power law noise. The power spectrum

% of the signal scales as f^(-alpha).

% Useage:

% x = powernoise(alpha, N)

% x = powernoise(alpha, N, 'option1', 'option2', ...)

% Inputs:

% alpha - power law scaling exponent

% N - number of samples to generate

% Output:

% x - N x 1 vector of power law samples

% With no option strings specified, the power spectrum is

% deterministic, and the phases are uniformly distributed in the range

% -pi to +pi. The power law extends all the way down to 0Hz (DC)

% component. By specifying the 'randpower' option string however, the

% power spectrum will be stochastic with Chi-square distribution. The

% 'normalize' option string forces scaling of the output to the range

% [-1, 1], consequently the power law will not necessarily extend

% right down to 0Hz.

% (cc) Max Little, 2008. This software is licensed under the

% Attribution-Share Alike 2.5 Generic Creative Commons license:

% http://creativecommons.org/licenses/by-sa/2.5/

% If you use this work, please cite:

% Little MA et al. (2007), "Exploiting nonlinear recurrence and fractal

% scaling properties for voice disorder detection", Biomed Eng Online, 6:23

% As of 20080323 markup

% If you use this work, consider saying hi on comp.dsp

% Dale B. Dalrymple

opt_randpow = false;

opt_normal = false;

for j = 1:(nargin-2)

switch varargin{j}

case 'normalize', opt_normal = true;

case 'randpower', opt_randpow = true;

end

end

N2 = floor(N/2)-1;

f = (2:(N2+1))';

A2 = 1./(f.^(alpha/2));

if (~opt_randpow)

p2 = (rand(N2,1)-0.5)*2*pi;

d2 = A2.*exp(i*p2);

else

% 20080323

p2 = randn(N2,1) + i * randn(N2,1);

d2 = A2.*p2;

end

d = [1; d2; 1/((N2+2)^alpha); flipud(conj(d2))];

x = real(ifft(d));

if (opt_normal)

x = ((x - min(x))/(max(x) - min(x)) - 0.5) * 2;

end

**Simulation Code B: Spiking neural network simulation code (MATLAB)**

function izhi_illustration_plosone(cop_val,detuning)

% Created by Eugene M. Izhikevich, February 25, 2003

% Modified by Eric Lowet, July, 2015

% For illustrative purposes:

% input

% cop_val = synaptic strength variable (mV) see line 59-51

% detuning = input strength difference between Network X and Y (E-cells) in

% mV

% Example: izhi_illustration_plosone(0.02,2)

Ne=800; Ni=200; % number of excitatory and inhibitory neurons

for trial=1

gampa=zeros(Ne,1,'single');

gaba=zeros(Ni,1,'single');

re=rand(Ne,1); ri=rand(Ni,1);

a=[0.017*ones(Ne,1); 0.1+0.02*ri]; % a =tiemscale of recover varibale u

b=[0.2+0.*re.*ones(Ne,1); 0.2+0.1*ri]; % b= sensitivity of u to subthreshold oscillations

c=[-65+0*re.^2; -65*ones(Ni,1)]; % c= membrane voltage after spike (reset)

d=[8*re.^2; 2*ones(Ni,1)]; % d= spike reset of recover varibale u

v=-65*ones(Ne+Ni,1); % Initial values of v = voltage

u=b.*v; % Initial values of u= membrane recovery variable

firings=[]; % spike timings

simulation_time=5000 ;

dt=1;

Ntot=(Ne+Ni); % Total number of neuron

Ne1=Ne/2;Ne2=Ne/2;

Ni1=Ni/2;Ni2=Ni/2;

%%%%%%%%%%%%% input strength gaussian input%%%%%%%%%%%%

% noise to neurons %%%%

var_E= 3.5; %

var_I= 3.5;

pr=10+((powernoise(1.5,simulation_time ))').*10^2; % for all E-cells of network: constant input + 1/f noise

pr2=10+detuning +((powernoise(1.5,simulation_time))').*10^2;

additional_input(:,[1:Ne1 Ne+1:Ne+Ni1])=repmat(pr,length([1:Ne1 Ne+1:Ne+Ni1]),1)';

additional_input(:,[Ne1+1:Ne1+Ne2 Ne+1+Ni1:Ntot]) =repmat(pr2,length([Ne1+1:Ne1+Ne2 Ne+1+Ni1:Ntot]),1)';

additional_input(:,Ne+1:Ntot)=0;

additional_input=additional_input';

%%%%%%%%%%%%%%%%%%%%

% synaptic decat constants

decay_ampa =2;

decay_gaba =8;

%% Constructing conenctivity matrix %%%%%%%

EE = 0.05 ;% ecitatory to excitatory

EI = 0.45;% ecitatory to inhibitory

IE = 0.5 ;% inhbitory to excitatory

II = 0.2;%2 % inhibitory to inhibitory

EE2 = 0.05;% ecitatory to excitatory

EI2 = 0.45 ;% ecitatory to inhibitory

IE2 = 0.5;% inhbitory to excitatory

II2 = 0.2;%2 % inhibitory to inhibitory

%cross-network conenctions

% E--> I

C1= cop_val; %E1->I2

C2= cop_val; %E2->I1

% E-->E

C3= cop_val/4;%E1->E2

C4= cop_val/4;%E2->E1

%%%%%%%%%%%%%%%%%%%%%%%%%%%%%%%%%%%%%%%%%

%%% WITHIN NETWORK CONNECTIVITY %%%%%%%%%

%%%%%%%%%%%%%%%%%%%%%%%%%%%%%%%%%%%%%%%%%

E1_ind=1:Ne1;E2_ind=Ne1+1:Ne1+Ne2;

I1_ind=Ne+1:Ne+Ni1;I2_ind=Ne+1+Ni1:Ne+Ni1+Ni2;

%%% E - E %%%%%%%%%

S(E1_ind,E1_ind) = EE*rand(Ne1);

S(E2_ind,E2_ind )= EE2*rand(Ne2);

%%% E - I %%%%%%%%%

S(I1_ind,E1_ind) = EI*rand(Ni1,Ne1);

S(I2_ind,E2_ind) = EI2*rand(Ni2,Ne2);

%%% I - E %%%%%%%%%

S( E1_ind ,I1_ind) = -IE*rand(Ne1,Ni1);

S( E2_ind ,I2_ind ) = -IE2*rand(Ne2,Ni2);

%%% I - I %%%%%%%%%

S( I1_ind,I1_ind ) = -II*rand(Ni1);

S( I2_ind,I2_ind ) =-II2*rand(Ni2);

%%%%%%%%%%%%%%%%%%%%%%%%%%%%%%%%%%%%%%%%%

%%% BETWEEN NETWORK CONNECTIVITY %%%%%%%%%

%%%%%%%%%%%%%%%%%%%%%%%%%%%%%%%%%%%%%%%%%

%%% E - I %%%%%%%%%

S( I2_ind, E1_ind ) =C2*rand(Ni2,Ne1);

S( I1_ind, E2_ind )= C1*rand(Ni1,Ne2);

%%% E - E %%%%%%%%%

S( E2_ind ,E1_ind ) =C3*rand(Ne2,Ne1);

S(E1_ind ,E2_ind ) =C4*rand(Ne1,Ne2);

%%%%%%%%%%%%%%%%%%%%%%%%%%%%%%%%%%%%%%%%%%

for t=1:dt:simulation_time % simulation

I=[var_E*randn(Ne,1);var_I*randn(Ni,1)]+additional_input(:,t); % thalamic input

fired=find(v>=30); % indices of spikes

firings=[firings; t+0*fired,fired];

gampa=gampa -gampa./decay_ampa;

gaba=gaba -gaba./decay_gaba;

gampa(v(1:Ne)>=30) =1;gaba(v(Ne+1:end)>=30) =1;

v(fired)=c(fired);

u(fired)=u(fired)+d(fired);

I=I+S*[gampa ;gaba]; % integrate input from other neurons

v=v+0.5*(0.04*v.^2+5*v+140-u+I); % step 0.5 ms

v=v+0.5*(0.04*v.^2+5*v+140-u+I); % for numerical

u=u+a.*(b.*v-u); % stability

end;

%%% spike probability for given time bin

excpop1=find(firings(:,2) >0 & firings(:,2) <= Ne1);

excpop2=find(firings(:,2) >Ne1 & firings(:,2) <= Ne);

inhpop1=find(firings(:,2) >Ne & firings(:,2) <= Ne+Ni1);

inhpop2=find(firings(:,2) >Ne+Ni1 & firings(:,2) <= Ne+Ni);

% population signal

[t1,t2] = hist(firings(excpop1,1),0:1:t);

signal1=fastsmooth(t1(300:end),3,3,1);

[t1,t2] = hist(firings(excpop2,1),0:1:t);

signal2=fastsmooth(t1(300:end),3,3,1);

% Filtering

Fn = 500;Fbp=[25 70];

[B, A] = butter(4, [min(Fbp)/Fn max(Fbp)/Fn]);

signal1= filtfilt(B,A,signal1);

signal2= filtfilt(B,A,signal2);

% Plotting

figure('Color','w','Position' ,[ 100 100 600 350]),

subplot(2,1,1,'Fontsize',15) %

firingexc=firings(excpop1,:);firinginh=firings(inhpop1,:);

plot(firingexc(:,1)./10^3,firingexc(:,2),'.','Color', [ 0.8 0.2 0.2]); % spike raster

hold on, plot(firinginh(:,1)./10^3,firinginh(:,2)-400,'.','Color', [ 0.2 0.2 0.8]);

axis tight;%set(gca,'xticklabel',[])

xlim([ 1 1.5]);title('Network X')

subplot(2,1,2,'Fontsize',15) %

firingexc=firings(excpop2,:);firinginh=firings(inhpop2,:);

plot(firingexc(:,1)./10^3,firingexc(:,2)-400,'.','Color', [ 0.8 0.2 0.2]); % spike raster

hold on, plot(firinginh(:,1)./10^3,firinginh(:,2)-500,'.','Color', [ 0.2 0.2 0.8]);

axis tight;%set(gca,'xticklabel',[])

xlim([ 1 1.5])

xlabel('Time (s) ');title('Network y')

p1=angle(hilbert((signal1) ));

p2=angle(hilbert((signal2)));

px=angle(exp(1i*p1)./exp(1i*p2)); % instant. phase-relation

figure('Color','w','Position' ,[ 200 200 250 220]),

subplot(1,1,1,'Fontsize',15) %

[ n,c]=hist(px,20);bar(c,n./sum(n))

h = findobj(gca,'Type','patch');

set(h,'FaceColor','k','EdgeColor', [ 0.3 0.3 0.3])

xlim([ -pi pi]);xlabel( 'phase-relation');ylabel('probability')

end

%%%%%% SUBFUNCTIONS %%%%%%%%%%%%%%%%%%%%%%

%%%%%%%%%%%%%%%%%%%%%%%%%%%%%%%%%%%%%%

function SmoothY=fastsmooth(Y,w,type,ends)

% fastbsmooth(Y,w,type,ends) smooths vector Y with smooth

% of width w. Version 2.0, May 2008.

% The argument "type" determines the smooth type:

% If type=1, rectangular (sliding-average or boxcar)

% If type=2, triangular (2 passes of sliding-average)

% If type=3, pseudo-Gaussian (3 passes of sliding-average)

% The argument "ends" controls how the "ends" of the signal

% (the first w/2 points and the last w/2 points) are handled.

% If ends=0, the ends are zero. (In this mode the elapsed

% time is independent of the smooth width). The fastest.

% If ends=1, the ends are smoothed with progressively

% smaller smooths the closer to the end. (In this mode the

% elapsed time increases with increasing smooth widths).

% fastsmooth(Y,w,type) smooths with ends=0.

% fastsmooth(Y,w) smooths with type=1 and ends=0.

% T. C. O'Haver, May, 2008.

if nargin==2, ends=0; type=1; end

if nargin==3, ends=0; end

switch type

case 1

SmoothY=sa(Y,w,ends);

case 2

SmoothY=sa(sa(Y,w,ends),w,ends);

case 3

SmoothY=sa(sa(sa(Y,w,ends),w,ends),w,ends);

end

function SmoothY=sa(Y,smoothwidth,ends)

w=round(smoothwidth);

SumPoints=sum(Y(1:w));

s=zeros(size(Y));

halfw=round(w/2);

L=length(Y);

for k=1:L-w,

s(k+halfw-1)=SumPoints;

SumPoints=SumPoints-Y(k);

SumPoints=SumPoints+Y(k+w);

end

s(k+halfw)=sum(Y(L-w+1:L));

SmoothY=s./w;

% Taper the ends of the signal if ends=1.

if ends==1,

startpoint=(smoothwidth + 1)/2;

SmoothY(1)=(Y(1)+Y(2))./2;

for k=2:startpoint,

SmoothY(k)=mean(Y(1:(2*k-1)));

SmoothY(L-k+1)=mean(Y(L-2*k+2:L));

end

SmoothY(L)=(Y(L)+Y(L-1))./2;

end

function x = powernoise(alpha, N, varargin)

% Generate samples of power law noise. The power spectrum

% of the signal scales as f^(-alpha).

% Useage:

% x = powernoise(alpha, N)

% x = powernoise(alpha, N, 'option1', 'option2', ...)

% Inputs:

% alpha - power law scaling exponent

% N - number of samples to generate

% Output:

% x - N x 1 vector of power law samples

% With no option strings specified, the power spectrum is

% deterministic, and the phases are uniformly distributed in the range

% -pi to +pi. The power law extends all the way down to 0Hz (DC)

% component. By specifying the 'randpower' option string however, the

% power spectrum will be stochastic with Chi-square distribution. The

% 'normalize' option string forces scaling of the output to the range

% [-1, 1], consequently the power law will not necessarily extend

% right down to 0Hz.

% (cc) Max Little, 2008. This software is licensed under the

% Attribution-Share Alike 2.5 Generic Creative Commons license:

% http://creativecommons.org/licenses/by-sa/2.5/

% If you use this work, please cite:

% Little MA et al. (2007), "Exploiting nonlinear recurrence and fractal

% scaling properties for voice disorder detection", Biomed Eng Online, 6:23

% As of 20080323 markup% If you use this work, consider saying hi on comp.dsp

% Dale B. Dalrymple

opt_randpow = false;

opt_normal = false;

for j = 1:(nargin-2)

switch varargin{j}

case 'normalize', opt_normal = true;

case 'randpower', opt_randpow = true;

end

end

N2 = floor(N/2)-1;

f = (2:(N2+1))';

A2 = 1./(f.^(alpha/2));

if (~opt_randpow)

p2 = (rand(N2,1)-0.5)*2*pi;

d2 = A2.*exp(i*p2);

else

p2 = randn(N2,1) + i * randn(N2,1);

d2 = A2.*p2;

end

d = [1; d2; 1/((N2+2)^alpha); flipud(conj(d2))];

x = real(ifft(d));

if (opt_normal)

x = ((x - min(x))/(max(x) - min(x)) - 0.5) * 2;

end
